# Discovery of TAK-925 as a Potent, Selective, and Brain-Penetrant Orexin 2 Receptor Agonist

**DOI:** 10.1021/acsmedchemlett.1c00626

**Published:** 2022-02-04

**Authors:** Tatsuhiko Fujimoto, Kentaro Rikimaru, Koichiro Fukuda, Hiromichi Sugimoto, Kei Masuda, Norio Ohyabu, Yoshihiro Banno, Norihito Tokunaga, Tetsuji Kawamoto, Yoshihide Tomata, Yasumi Kumagai, Motoo Iida, Yoichi Nagano, Mariko Yoneyama-Hirozane, Yuji Shimizu, Katsunori Sasa, Takashi Ishikawa, Hiroshi Yukitake, Mitsuhiro Ito, Kazunobu Aoyama, Takahiro Matsumoto

**Affiliations:** Research, Takeda Pharmaceutical Company Limited, 26-1, Muraoka-Higashi 2-Chome, Fujisawa, Kanagawa 251-8555, Japan

**Keywords:** Orexin 2 receptor agonist, OX2R, TAK-925

## Abstract

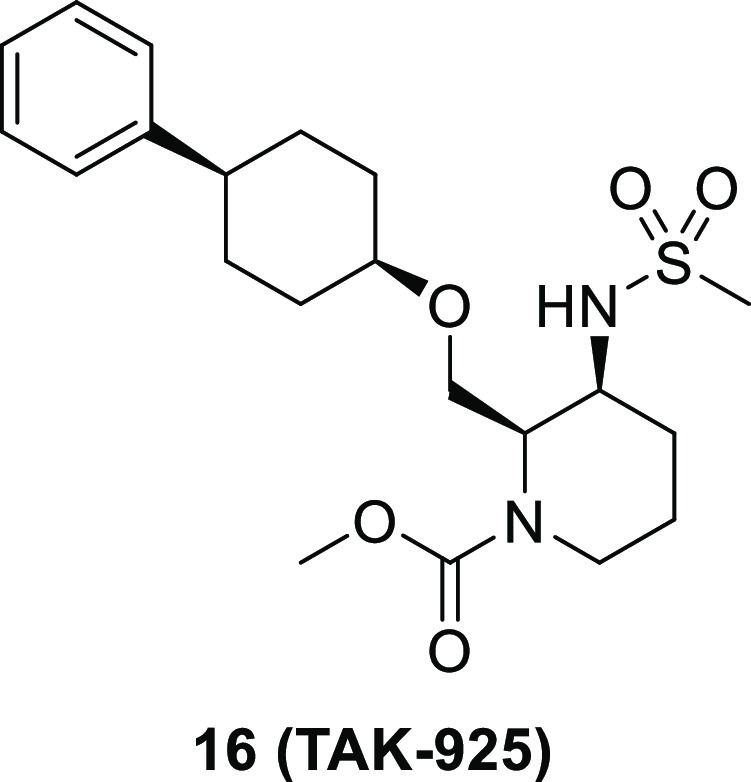

TAK-925, a potent, selective, and brain-penetrant orexin 2 receptor (OX2R) agonist, [methyl (2*R*,3*S*)-3-((methylsulfonyl)amino)-2-(((*cis*-4-phenylcyclohexyl)oxy)methyl)piperidine-1-carboxylate, **16**], was identified through the optimization of compound **2**, which was discovered by a high throughput screening (HTS) campaign. Subcutaneous administration of compound **16** produced wake-promoting effects in mice during the sleep phase. Compound **16** (TAK-925) is being developed for the treatment of narcolepsy and other related disorders.

Orexin A (OX-A) and orexin B (OX-B) are the hypothalamic neuropeptides which are known as important regulators of sleep/wakefulness states.^1,2^ Loss of orexinergic neurons in the brain is associated with the cause of narcolepsy type 1 (NT1) characterized by excessive daytime sleepiness, cataplexy, hypnagogic/hypnopompic hallucinations, sleep paralysis, and disturbed nighttime sleep.^3−6^

Orexin neuropeptides exert their effects through the activation of the G protein-coupled receptors identified as orexin receptor type 1 (OX1R) and type 2 (OX2R). OX2R knockout (KO) mice exhibit apparent narcolepsy-like phenotypes including fragmentation of sleep/wakefulness and cataplexy-like episodes, while OX1R KO mice do not show significant behavioral abnormalities.^7,8^ Thus, OX2R activation is anticipated to be a promising therapeutic option for NT1.

Since the endogenous orexin peptides cannot efficiently penetrate the blood-brain barrier (BBB),^9^ brain-penetrant and small-molecule orexin agonists would be attractive for the treatment of the sleep-related disorders including NT1.^10^

YNT-185 (Figure 1) was reported as the first nonpeptide OX2R agonist.^11^ The analogous compound **1**(12) was recently published with its cocomplex structure with active-state OX2R obtained by cryogenic electron microscopy (cryo-EM). The analogous compounds with OX1R/OX2R agonistic activities have been also disclosed.^13^ However, structurally different OX2R agonists with smaller molecular weights compared with those in this series (YNT-185: 616, compound **1**: 624) should be explored to develop brain-penetrant therapeutic OX2R agonist.^14^

**Figure 1:**
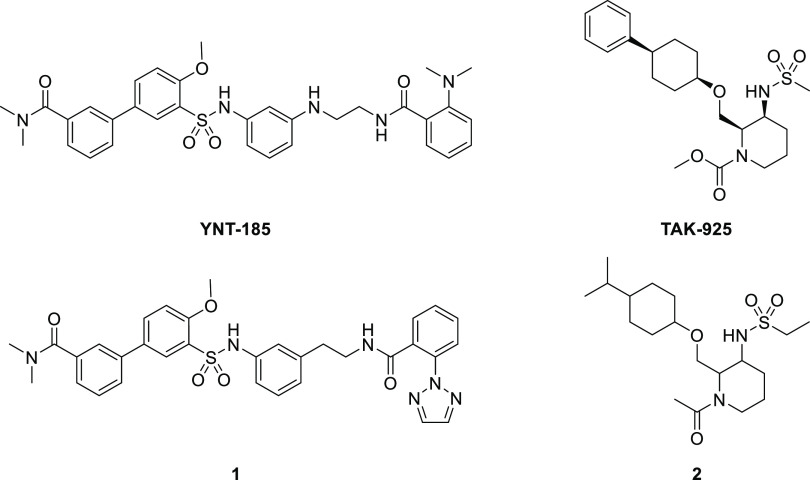
Chemical structures of reported OX2R agonists and our hit compound **2**.

Recently, we reported that TAK-925 (Figure 1), developed as a potent and selective agonist for OX2R, shows a therapeutic potential for diseases associated with hypersomnia in mice.^15^ TAK-925 has been investigated as a drug for the treatment of hypersomnia including NT1 (Clincaltrials.gov Registry Identifier: NCT03332784). In this paper, we report the design, synthesis, and discovery of brain-penetrant small molecule OX2R agonist TAK-925 starting from a high throughput screening (HTS) campaign, followed by the optimization of hit compound.

An HTS campaign to discover OX2R agonists was performed by measuring calcium flux as a functional determinant of OX2R agonism using a fluorometric imaging plate reader (FLIPR) assay system. As a result, the hit compound **2** (diastereomeric mixtures, EC_50_ = 570 nM, maximum response [*E*_max_] = 94%) was identified with moderate EC_50_ but full OX2R agonistic activity comparable to OX-A (Figure 1). Compound **2** exhibited good selectivity against OX1R agonism (EC_50_ > 100 000 nM), as well as the good characteristics for central nervous system (CNS) drugs such as smaller molecular weight (389) and favorable topological polar surface area (TPSA, TPSA = 76), indicating that compound **2** is a promising starting point for the development of selective and brain-penetrant OX2R agonist drug candidates.

Our hit compound **2** was a diastereomeric mixture, thus our first effort was an evaluation of all possible diastereomers (**3**–**6**; Table 1). Among four *cis* and *trans* isomers for each ring A and B, *cis*-*cis* derivative **3** showed the most potent OX2R agonistic activity (EC_50_ = 270 nM). In addition, compound **3** maintained a good selectivity against OX1R agonism (EC_50_ > 100 000 nM). These results imply that the OX2R receptor strongly recognizes the compound stereochemistry.

**Table 1:**
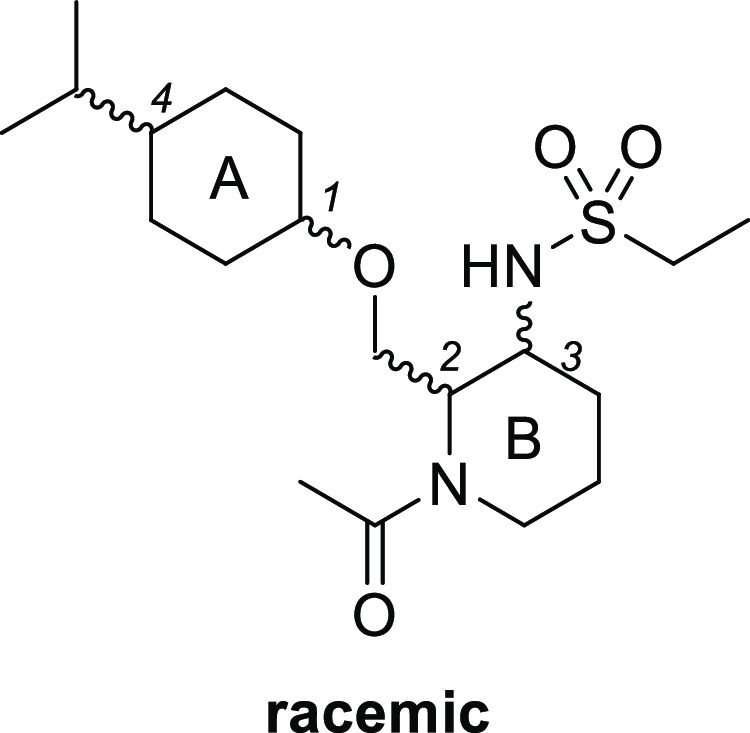
In Vitro Activities of Compounds **3**–**6**

			EC_50_ (nM)^a^	
compd	ring A 1,4-position	ring B 2,3-position	OX2R agonistic activity^b^	OX1R agonistic activity^b^
**3**	*cis*	*cis*	270 (250–300)	>100 000
**4**	*cis*	*trans*	>30 000	>100 000
**5**	*trans*	*cis*	>30 000	>100 000
**6**	*trans*	*trans*	>30 000	>100 000
OX-A			0.18 (0.15–0.21)	0.068 (0.053–0.086)

a EC_50_ values and 95% confidence intervals were calculated from duplicate measurements. All values are rounded to two significant digits. *n* = 2.

b Calcium flux assay with Chinese hamster ovary cells expressing human OX2R or human OX1R.

We conducted optimization of the sulfonamide, isopropyl, and carbonyl parts of compound **3** to increase the OX2R agonistic activity (Figure 2).

**Figure 2:**
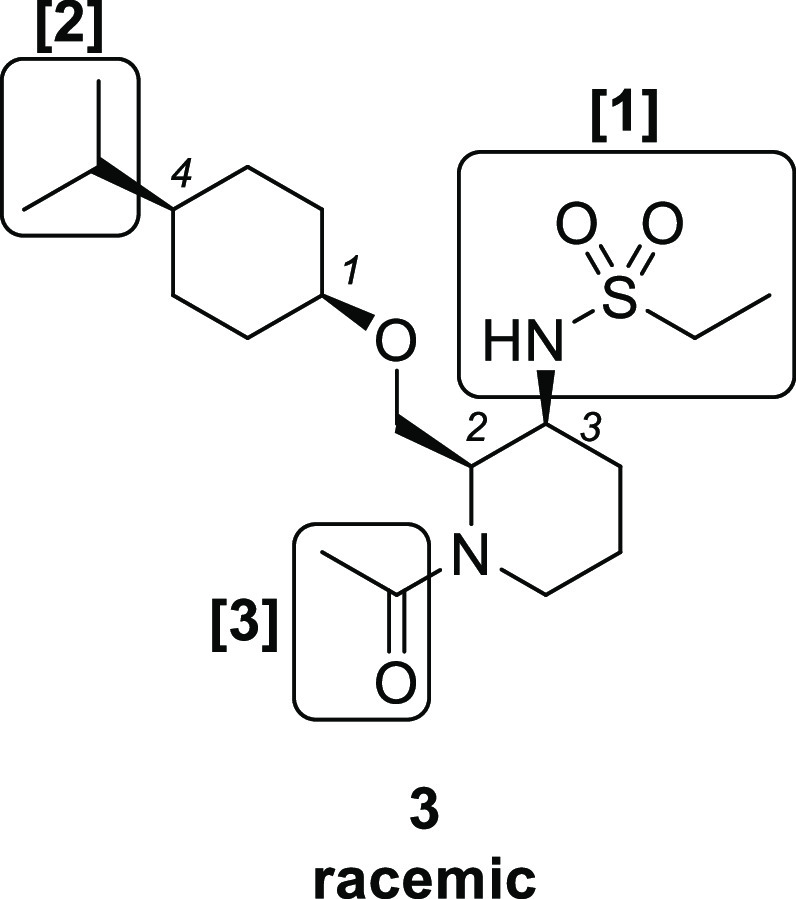
Lead optimization of compound **3**.

We first examined the modification of the sulfonamide part of compound **3** (Table 2). Replacement of the ethyl group of compound **3** with a methyl group maintained the OX2R agonistic activity (**7**, EC_50_ = 330 nM), whereas the isopropyl (**8**, EC_50_ = 940 nM) and propyl (**9**, EC_50_ = 2500 nM) derivatives showed moderate activity. Methylation of nitrogen atoms in the sulfonamide (**10**, EC_50_ > 30 000 nM) or replacement of sulfonamide with acetamide (**11**, EC_50_ > 30000 nM) reduced agonist activity, indicating that the secondary sulfonamide may play an important role in OX2R agonistic activity. Considering the ligand lipophilicity efficiency^16,17^ (LLE, LLE = (pEC_50_) – clogP, clogP: calculated using ChemDraw) of these compounds as a drug likeness index, compound **7** (LLE = 3.5, clogP = 3.0), which showed higher value than compound **3** (LLE = 3.0, clogP = 3.6) was selected as a lead compound for further exploration.

**Table 2:**
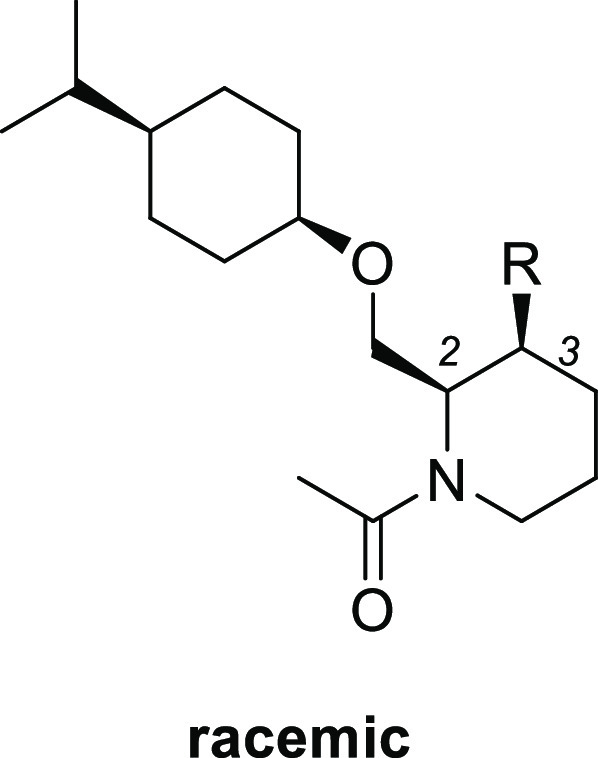

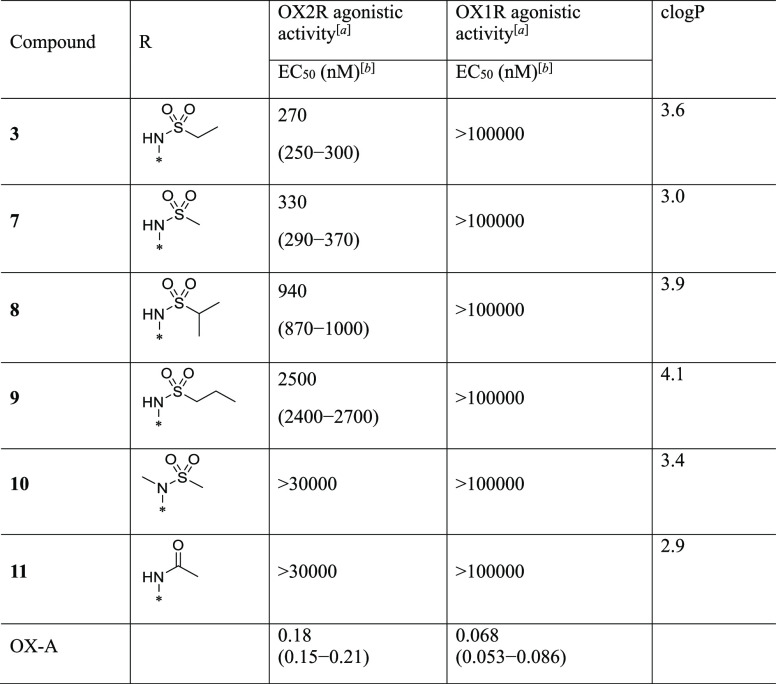
In Vitro Activities of Compounds **3** and **7–11**

a Calcium flux assay with Chinese hamster ovary cells expressing human OX2R or human OX1R.

b EC_50_ values and 95% confidence intervals were calculated from duplicate measurements. All values are rounded to two significant digits. *n* = 2.

We next explored the substituent at the 4-position of the cyclohexane ring (Table 3). Compared with isopropyl compound **7**, smaller methyl compound **12** showed decreased activity (EC_50_ = 3700 nM). On the other hand, the larger phenyl compound **13** increased the potency (EC_50_ = 140 nM). Chiral separation of compound **13** afforded the corresponding enantiomer pairs **13a** (EC_50_ = 66 nM) and **13b** (EC_50_ = > 30000 nM).

**Table 3:**
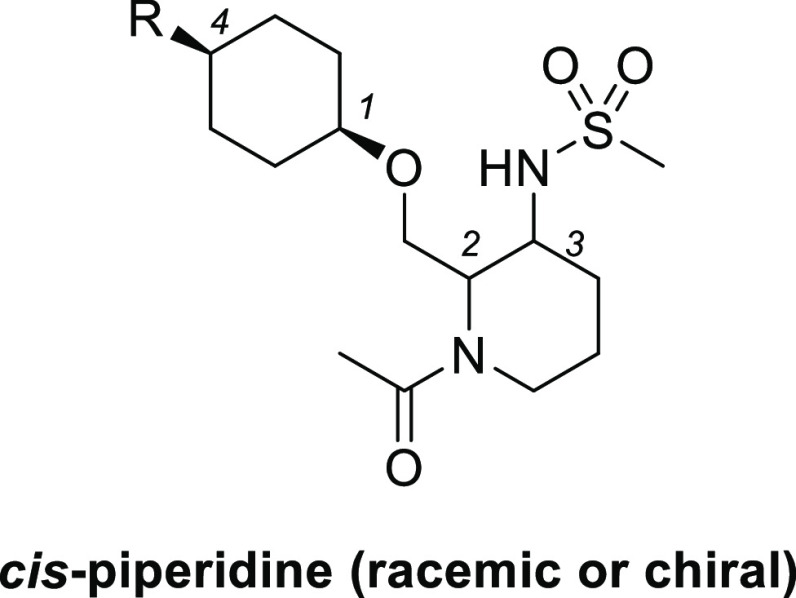

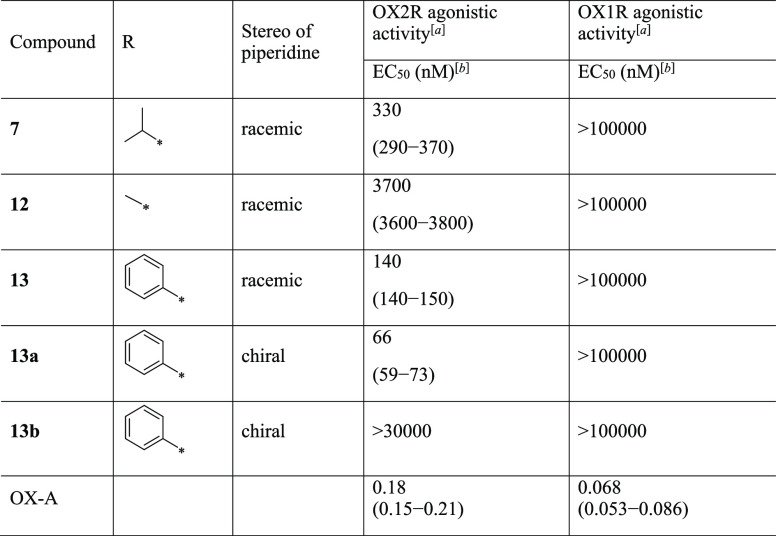
In Vitro Activities of Compounds **7**, **12**, **13**, **13a**, and **13b**

a Calcium flux assay with Chinese hamster ovary cells expressing human OX2R or human OX1R.

b EC_50_ values and 95% confidence intervals were calculated from duplicate measurements. All values are rounded to two significant digits. *n* = 2.

Finally, substituents at the 1-position of piperidine were explored starting from chiral compound **13a** (Table 4). Removal of acetamide resulted in a large reduction in agonistic activity (**14**, EC_50_ = 2500 nM), suggesting that the carbonyl group is a key factor in potent OX2R agonistic activity. Elongation of the acetyl group to propionyl increased the activity (**15**, EC_50_ = 11 nM). Carbamate derivative **16** (EC_50_ = 5.5 nM) also exhibited better potency than compound **15**. Further elongation of the alkyl group in compound **16** from methyl to ethyl led to the decreased potency (**17**, EC_50_ = 27 nM). On the other hand, conversion of the carbamate moiety of **18** with urea increased the activity up to subnanomolar range (**18**, EC_50_ = 0.29 nM), which is comparable to the agonist activity of endogenous OX-A peptide in our calcium flux assay system. We also measured the multidrug resistance protein 1 (MDR-1) efflux ratio of these chiral compounds, as an index for blood-brain permeability.^18^ Among these compounds, we selected carbamate compound **16**(19) with the best balanced profile for further evaluation. Compound **16** (molecular weight: 425) exhibited good potency (OX2R EC_50_: 5.5 nM), selectivity (OX1R EC_50_: > 100 000 nM), and MDR-1 efflux ratio (0.8, A to B = 169). Compound **16** also showed good selectivity against 106 off-target enzymes and receptors.^15^

**Table 4:**
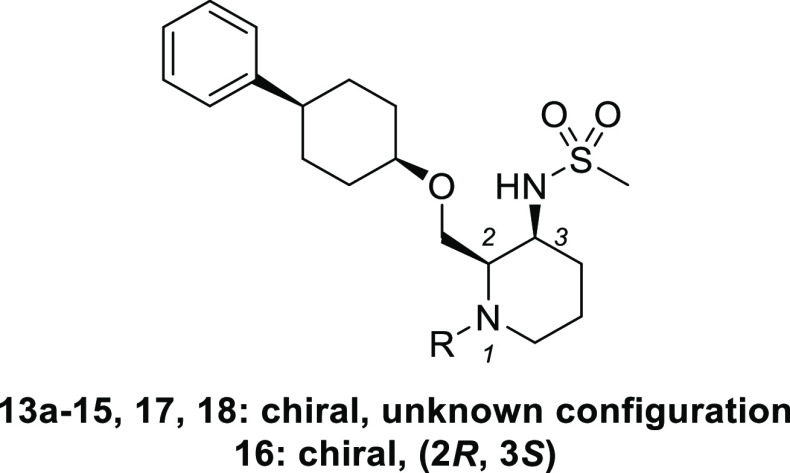

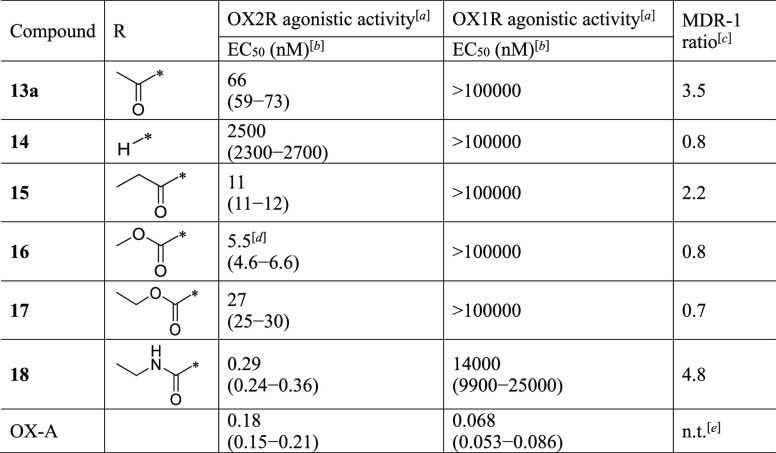
In Vitro Activities of Compounds **13a**, **14**–**18**, and OX-A

a Calcium flux assay with Chinese hamster ovary cells expressing human OX2R or human OX1R.

b EC_50_ values and 95% confidence intervals were calculated from duplicate measurements. All values are rounded to two significant digits. *n* = 2.

c MDR-1 directional transport ratio (B to A/A to B).

d *n* = 4.

e n.t. = not tested.

X-ray and nuclear magnetic resonance (NMR) conformational analysis of newly discovered OX2R agonists were performed to reveal the optimal conformation to show OX2R agonism (Figures 3 and 4). X-ray crystal data of highly active compound **16** (EC_50_ = 5.5 nM) revealed that both the methylene linker at the 2-position of the (2*R*,3*S*)-piperidine and the ether linker at the 1-position of the cyclohexane formed axial orientations (Figure 3). This conformation was also supported by the NMR nuclear Overhauser effect spectroscopy (NOESY) spectra of compound **16**. Conformational analysis of compound **5** (EC_50_ > 30 000 nM) and compound **14** (EC_50_ = 2500 nM), which showed lower activity than compound **16**, were also conducted by NMR NOESY studies. Compound **5** formed a chair conformation with equatorial substituent at the 1-position of the cyclohexane, while it maintained axial orientation at the 2-position of the piperidine (Figure 4). This result indicates that the axial methylene linker at the 1-position of the cyclohexane plays a crucial role in the OX2R agonistic activity. In addition, compound **14** has the equatorial substituent at the 2-position of the piperidine, keeping axial orientation at the 1-position of the cyclohexane, indicating that the axial state on the piperidine is also important for the OX2R agonistic activity. Thus, it was suggested that the unique but stable axial–axial conformation between piperidine and the cyclohexane ring of compound **16** was favorable for the OX2R agonistic activity.

**Figure 3:**
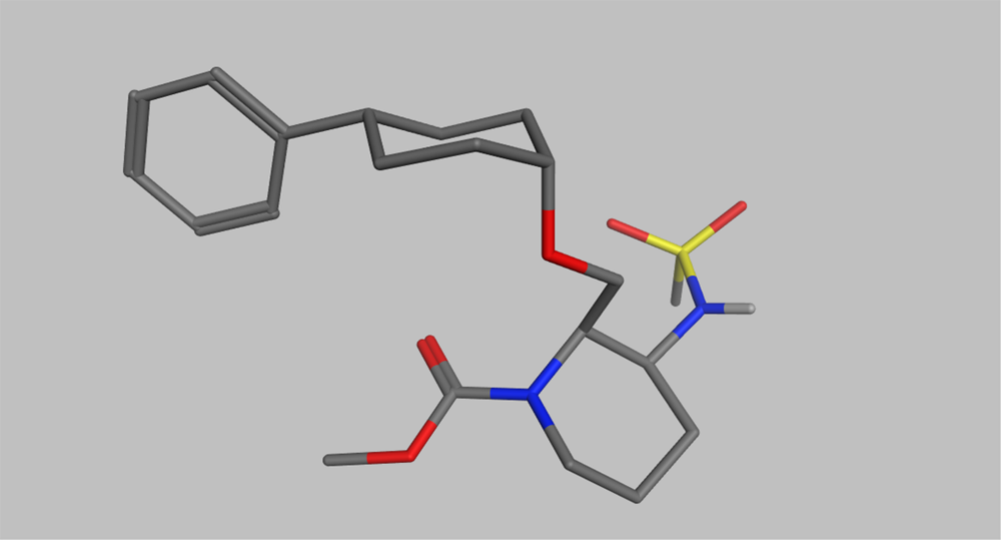
X-ray crystal structure of compound **16**.

**Figure 4:**
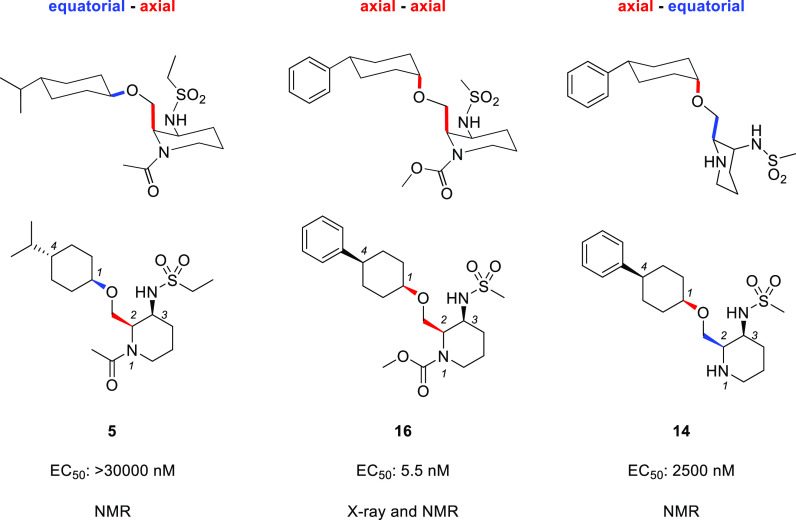
Conformational analysis of compounds **5**, **16**, and **14**.

Brain and plasma concentration of compound **16** was measured after intraperitoneal administration at 10 mg/kg in mice (Table 5). Although compound **16** showed short half-life in mice, **16** showed acceptable brain-to-plasma concentration ratio (0.2) at 0.5 and 1 h after administration respectively, indicating that compound **16** is brain-penetrant.

**Table 5:** Brain Concentration of Compound **16** in Mice at 10 mg/kg, ip^a^

time (h)	brain concn (ng/g)	plasma concn (ng/mL)	brain-to-plasma concn ratio
0.5	183	880	0.2
1	63	288	0.2

a C57BL/6J mice, intraperitoneal administration, *n* = 3.

It has been reported that activation of the orexin system by intracerebroventricular injection of OX-A increases wakefulness in rodents.^20,21^ In this study, we assessed the effect of compound **16** on wakefulness time in ICR mice during the sleep phase based on the measurements of electroencephalogram and electromyogram. Subcutaneous administration of compound **16** at 3 mg/kg significantly increased total wakefulness time during 3 h after administration in ICR mice (Figure 5). These results demonstrate that compound **16** is brain-penetrant and shows arousal effect in mice.

**Figure 5:**
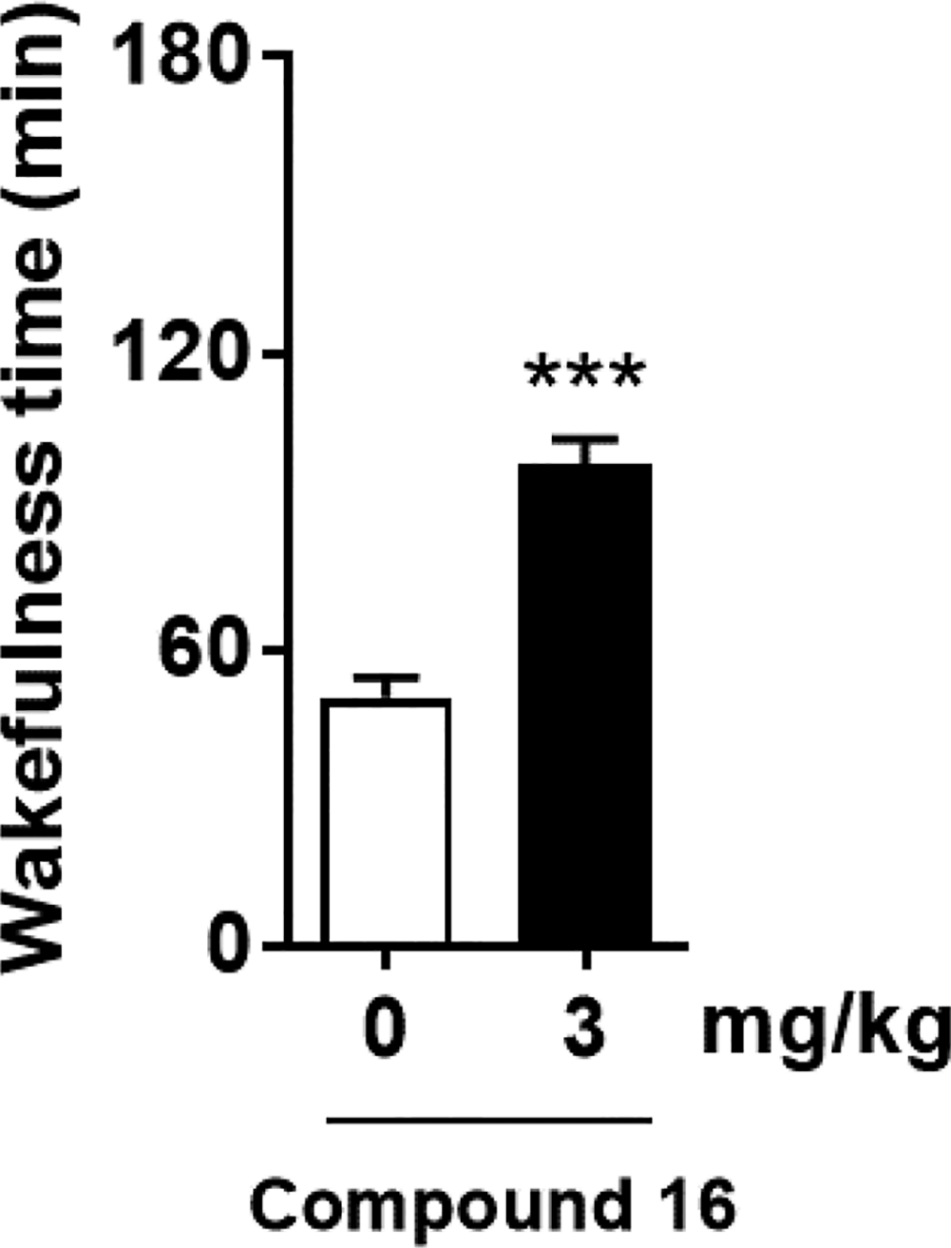
Effect of compound **16** on wakefulness time in ICR mice during the sleep phase. Compound **16** at 3 mg/kg or vehicle was administered subcutaneously to ICR mice at zeitgeber time 5, and electroencephalograms and electromyograms were recorded. Analysis was performed with data collected during 3 h after drug administration. Data were presented as the mean + standard error of the mean (*n* = 8). ***P < 0.001, compared with the vehicle-treated mice (two-tailed paired *t* test).

We have developed a potent, selective, and brain-penetrant OX2R agonist, compound **16**, starting from a hit compound **2**. Conformational analysis of compound **16** revealed the unique axial–axial conformation, which might contribute to the potent OX2R agonistic activity. Subcutaneous administration of compound **16** significantly increased total wakefulness time in mice during the sleep phase. Compound **16** (TAK-925) is a promising therapeutic agent as an OX2R agonist for the treatment of narcolepsy and other related disorders.

## Supporting Information Available

Analytical HPLC traces (compound purity in HPLC); chemical synthesis procedures; calcium flux assay (in vitro agonistic activity of OX2R and OX1R); transcellular transport study using a transporter-expression system; measurement of plasma and brain concentration of compound **16** in mice; evaluation of wakefulness time measurement of compound **16** in mice; ^1^H NMR and ^13^C NMR chart of compound **16**; single-crystal X-ray structure analysis of compound **16** (PDF)
