# Supplementary material for: Discovery of TAK-925 as a Potent, Selective, and Brain-Penetrant Orexin 2 Receptor Agonist
Source: ACS Med Chem Lett. 2022 Feb 4;13(3):457–62. doi: 10.1021/acsmedchemlett.1c00626 (PMC8919389; doi:10.1021/acsmedchemlett.1c00626)
Supplement: Supplementary file 1 — ml1c00626_si_001.pdf [file ml1c00626_si_001.pdf]

Discovery of TAK-925 as a Potent, Selective and Brain-Penetrant Orexin 2 Receptor Agonist

Tatsuhiko Fujimoto,†Kentaro Rikimaru,†Koichiro Fukuda,†Hiromichi Sugimoto,†Kei Masuda,† Norio Ohyabu,† Yoshihiro Banno,†Norihito Tokunaga,† Tetsuji Kawamoto,†Yoshihide Tomata,† Yasumi Kumagai,†Motoo Iida,† Yoichi Nagano,† Mariko Yoneyama-Hirozane,† Yuji Shimizu,† Katsunori Sasa,† Takashi Ishikawa,† Hiroshi Yukitake,† Mitsuhiro Ito,†Kazunobu Aoyama,†and Takahiro Matsumoto†*

† Research, Takeda Pharmaceutical Company Limited, 26-1, Muraoka-Higashi 2-Chome, Fujisawa Kanagawa 251-8555, Japan

Corresponding author

Norihito Tokunaga Ph.D.

Research, Takeda Pharmaceutical Company Limited, 26-1 Muraoka-Higashi 2-Chome, Fujisawa, Kanagawa 251-8555, Japan

Phone No: +81-466-32-1218

Email Address: norihito.tokunaga@takeda.com

ORCID: 0000-0002-5693-6858

Table of contents

Analytical HPLC traces (compound purity in HPLC) **S3**

Chemical synthesis procedures **S9**

Calcium flux assay (in vitro agonistic activity of OX2R and OX1R) **S36**

Transcellular transport study using a transporter-expression system **S37**

Measurement of plasma and brain concentration of compound 16 in mice **S38**

Evaluation of wakefulness time measurement of compound 16 in mice **S39**

^{1}H NMR and ^{13}C NMR charts of compound 16 **S40**

Single-crystal X-ray structure analysis of compound 16 **S41**

References **S42**

Analytical HPLC traces (compound purity in HPLC)

Analytical HPLC method. Analytical HPLC was performed with Corona Charged Aerosol Detector (CAD), Nano quantity analyte detector (NQAD), or photo diode array detector. The column was a Capcell Pak C18AQ (50 mm × 3.0 mm I.D., Shiseido, Japan) or L-column 2 ODS (30 mm × 2.0 mm I.D., CERI, Japan) with a temperature of 50 °C and a flow rate of 0.5 mL/min. Mobile phase A and B under a neutral condition were a mixture of 50 mmol/L ammonium acetate, water, and acetonitrile (1:8:1, v/v/v) and a mixture of 50 mmol/L ammonium acetate and acetonitrile (1:9, v/v), respectively. The ratio of mobile phase B was increased linearly from 5% to 95% over 3 min, 95% over the next 1 min. Mobile phase A and B under an acidic condition were a mixture of 0.2% formic acid in 10 mmol/L ammonium formate and 0.2% formic acid in acetonitrile, respectively. The ratio of mobile phase B was increased linearly from 14% to 86% over 3 min, 86% over the next 1 min. The purities of all tested compounds were >95% as determined by elemental analyses within ±0.4% of the calculated values or analytical HPLC.

Compound 3

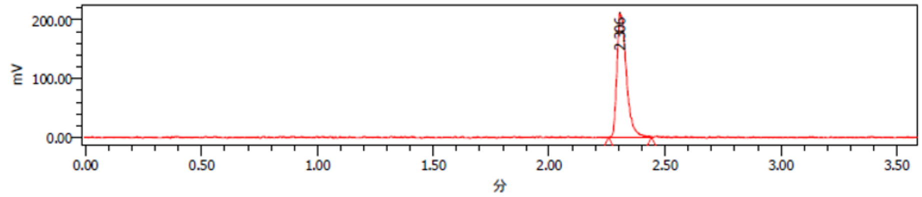

	tR (分)	Area (μV秒)	%Area
1	2.334	694020	100.00

Compound 4

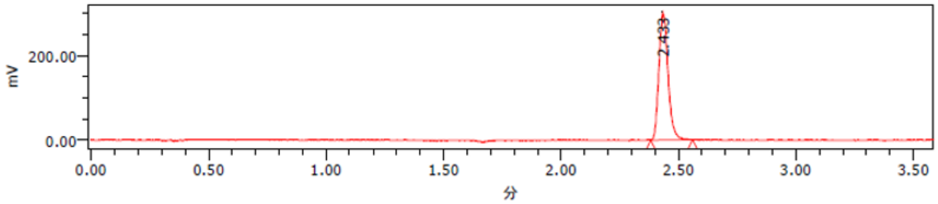

	tR (分)	Area (μV秒)	%Area
1	2.433	824174	100.00

Compound 5

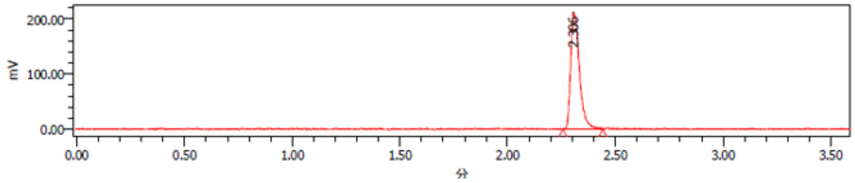

	tR (分)	Area (μV秒)	%Area
1	2.306	599429	100.00

Compound 6

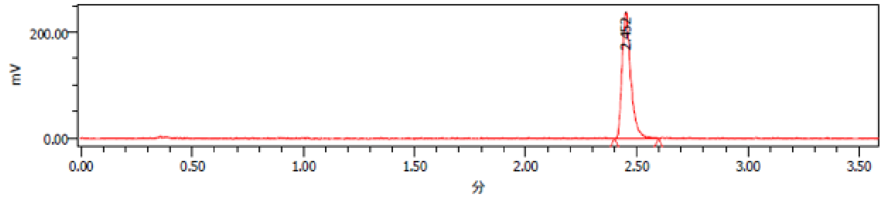

	tR (分)	Area (μV秒)	%Area
1	2.452	640253	100.00

Compound 7

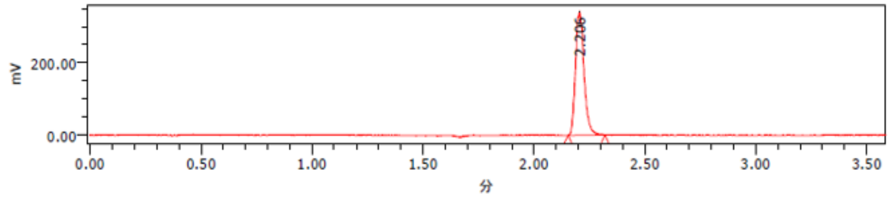

	tR (分)	Area (μV秒)	%Area
1	2.206	908748	100.00

Compound 8

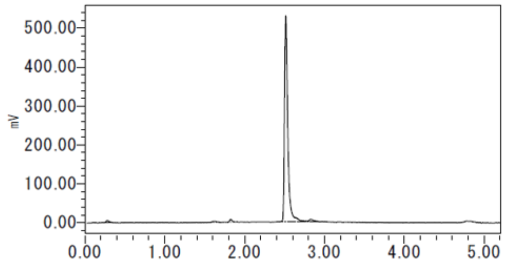

Result of Analysis

	Retention Time (min)	Peak area (μV×sec)	Divisional method	% of Area
1	0.286	14891	bb	0.94
2	1.621	9517	bb	0.60
3	1.826	15522	BB	0.98
4	2.516	1510570	BB	95.76
5	2.643	4112	tt	0.26
6	2.831	22855	TT	1.45

Compound 9

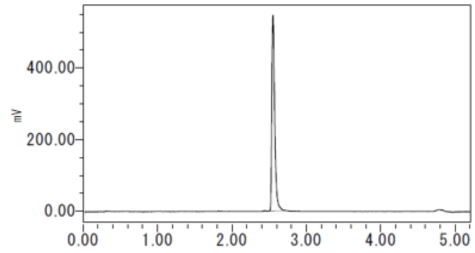

Result of Analysis

	Retention Time (min)	Peak area (μV×sec)	Divisional method	% of Area
1	2.443	3997	bb	0.27
2	2.552	1456093	BB	99.73

Compound 10

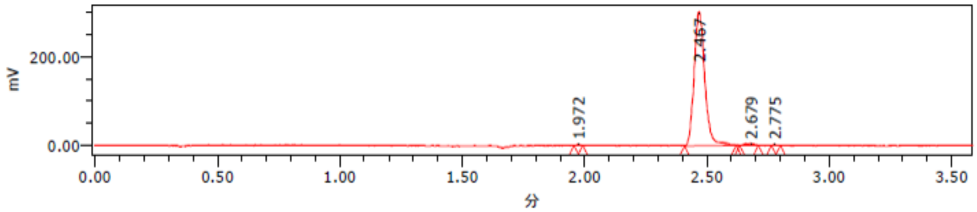

	tR (分)	Area (μV秒)	%Area
1	1.972	2800	0.30
2	2.467	911859	98.36
3	2.679	9551	1.03
4	2.775	2893	0.31

Compound 11

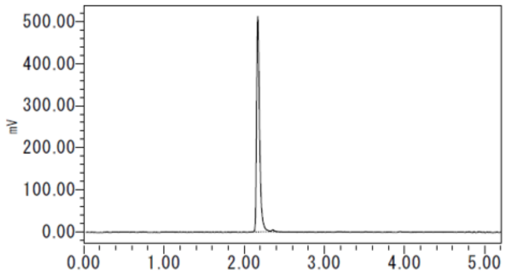

Result of Analysis

	Retention Time (min)	Peak area (μV×sec)	Divisional method	% of Area
1	2.171	1336868	BB	99.46
2	2.359	7307	TT	0.54

Compound 12

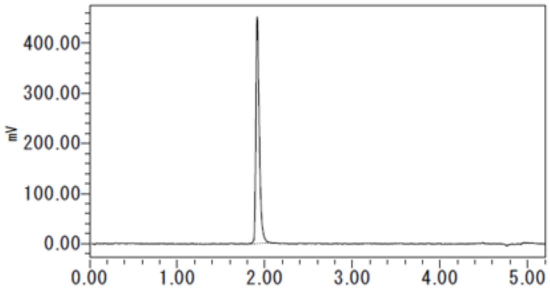

Result of Analysis

	Retention Time (min)	Peak area (μV×sec)	Divisional method	% of Area
1	1.914	1246586	VB	100.00

Compound 13

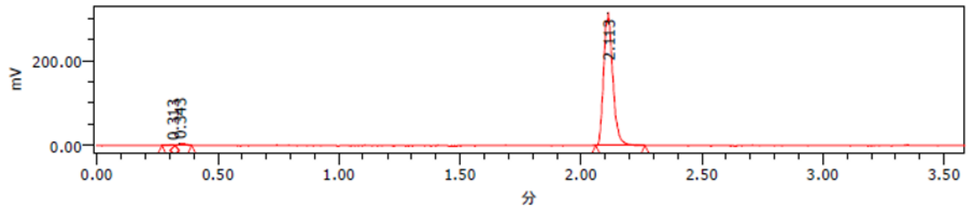

	tR (分)	Area (μV秒)	%Area
1	0.313	3184	0.38
2	0.343	10632	1.27
3	2.113	822293	98.35

Compound 13a

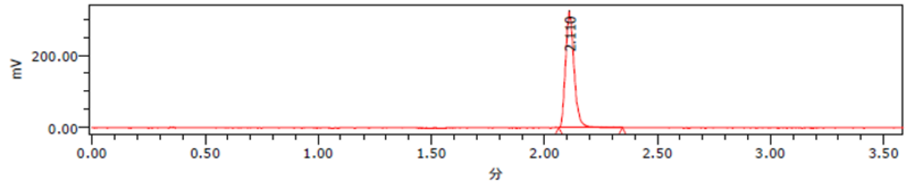

	tR (分)	Area (μV秒)	%Area
1	2.110	851075	100.00

Compound 13b

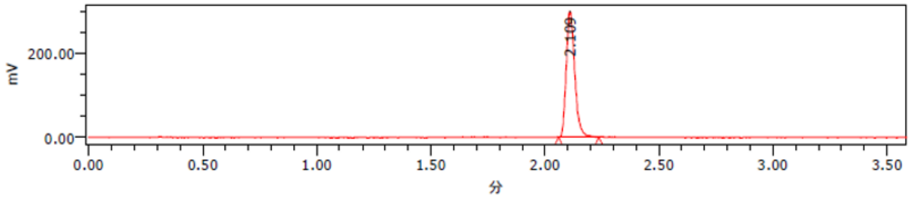

	tR (分)	Area (μV秒)	%Area
1	2.109	772149	100.00

Compound 14

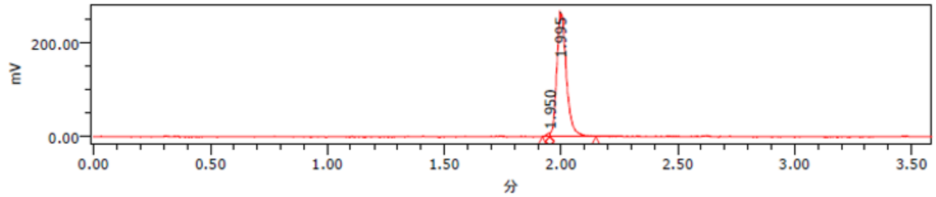

	tR (分)	Area (μV秒)	%Area
1	1.950	7784	1.02
2	1.995	756145	98.98

Compound 15

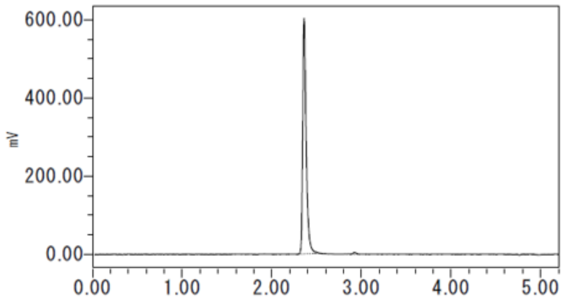

Result of Analysis

	Retention Time (min)	Peak area (μV × sec)	Divisional method	% of Area
1	2.361	1621603	BB	99.31
2	2.922	11290	BB	0.69

Compound 16

1)

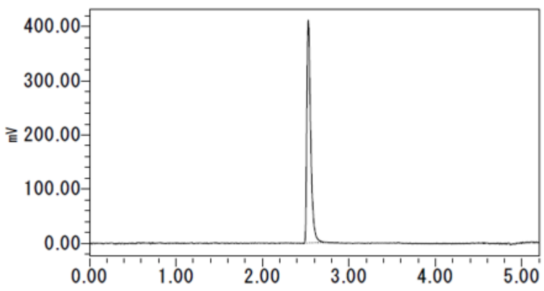

Result of Analysis

	Retention Time (min)	Peak area (μV × sec)	Divisional method	% of Area
1	2.530	1275780	BB	100.00

2)

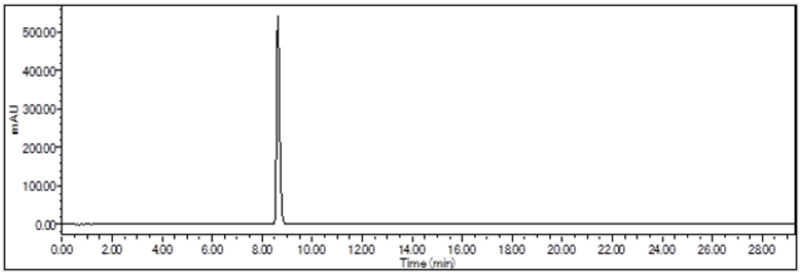

	保持時間	面積	%面積	高さ
1	1.232	3373	0.07	1295
2	1.429	519	0.01	164
3	7.608	421	0.01	59
4	8.629	4586898	99.89	541050
5	14.487	316	0.01	91
6	17.362	303	0.01	151

Instrument: LC-20A system (detector SPD M20A) (Shimadzu)
Column: SunShell C18, 150×4.6 mm i.d., 2.6 μm (ChromanicTechnologies)
Detection UV 215 nm
Mobile phase A: 0.05 mol/L sodium perchlorate buffer (adjusted to pH2.5 with perchloric acid aqueous solution)
Mobile phase B: Acetonitrile
Gradient:

Time (min)	%B
0	50
10	50
15	90
20	90
20.1	50
29.3	50

Column temp.: 35°C
Flow rate: 1.0 mL/min
Sample conc.: 200 μg/mL (Distilled water : acetonitrile = 1 : 1)
Injection vol.: 30 μL

Compound 17

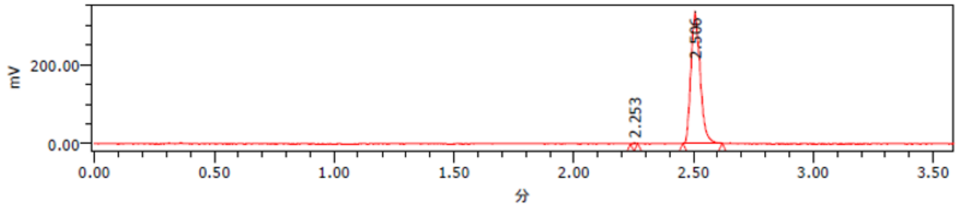

	tR (分)	Area (μV秒)	%Area
1	2.253	2018	0.23
2	2.506	893408	99.77

Compound 18

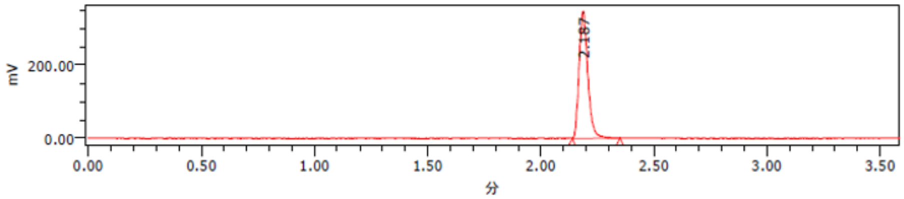

	tR (分)	Area (μV秒)	%Area
1	2.187	924168	100.00

Chemical synthesis procedures

General Chemistry Information. All solvents and reagents were obtained from commercial sources and were used as received. Microwave-assisted reactions were carried out in a single-mode reactor, Biotage Initiator 2.0 or 2.5 microwave synthesizer. Yields were not optimized. All reactions were monitored by thin layer chromatography (TLC) analysis on Merck Kieselgel 60 F254 plates or Fuji Silysia NH plates or liquid chromatography−mass spectrometry (LC−MS) analysis. LC−MS analysis was performed on a Shimadzu LC−MS system operating in APCI (+ or −) or ESI (+ or −) ionization mode. Analytes were eluted using a linear gradient with a mobile phase of water/acetonitrile containing 0.05% TFA or 5 mM ammonium acetate and detected at 220 nm. Column chromatography was carried out on silica gel ((Merck Kieselgel 60, 70−230 mesh, Merck) or (Chromatorex NH-DM 1020, 100−200 mesh, Fuji Silysia Chemical, Ltd.)) or on prepacked Purif-Pack columns (SI or NH, particle size 60 μm; Fuji Silysia Chemical, Ltd.). Analytical high-performance liquid chromatography (HPLC) was performed using a Corona charged aerosol detector or photodiode array detector with a Capcell Pak C18AQ (3.0 mm ID × 50 mm L, Shiseido, Japan) or L-column2 ODS (2.0 mm ID × 30 mm L, CERI, Japan) column at a temperature of 50 °C and a flow rate of 0.5 mL/min. Mobile phases A and B under neutral conditions were a mixture of 50 mmol/L ammonium acetate, water, and acetonitrile (1:8:1, v/v/v) and a mixture of 50 mmol/L ammonium acetate and acetonitrile (1:9, v/v), respectively. The ratio of mobile phase B was increased linearly from 5% to 95% over 3 min and then maintained at 95% over the next 1 min. Mobile phases A and B under acidic conditions were a mixture of 0.2% formic acid in 10 mmol/L ammonium formate and 0.2% formic acid in acetonitrile, respectively. The ratio of mobile phase B was increased linearly from 14% to 86% over 3 min and then maintained at 86% over the next 1 min. All final test compounds were purified to >95% chemical

purity as measured by analytical HPLC. Elemental analyses were carried out by Takeda Analytical Laboratories, and all results were within ±0.4% of the theoretical values. Melting points (mp) were determined on a Büchi B-545 melting point apparatus or a DSC1 system (Mettler-Toledo International Inc., Greifensee, Switzerland). The specific rotatory power was determined by the polarimeter (P-2300 Digital Polarimeter, JASCO corporation). Proton nuclear magnetic resonance (^{1}H NMR) spectra were recorded on a Varian Mercury-300 (300 MHz), Varian (400 MHz), Bruker DPX300 (300 MHz), Bruker Avance III (400 MHz), or Bruker AVANCE II^{+} (600 MHz) instruments. All ^{1}H NMR spectra were consistent with the proposed structures including a mixture of rotamers. All proton shifts are given in parts per million (ppm) downfield from tetramethysilane (δ) as the internal standard in deuterated solvent, and coupling constants (*J*) are in hertz (Hz). NMR data are reported as follows: chemical shift, integration, multiplicity (s, singlet; d, doublet; t, triplet; q, quartet; quint, quintet; spt, septet; m, multiplet; dd, doublet of doublets; td, triplet of doublets; ddd, doublet of doublet of doublets; brs, broad singlet; brd, broad doublet; brt, broad triplet; brquin, broad quintet; and brdd, broad doublet of doublets), and coupling constants. Very broad peaks for protons of, for example, hydroxyl and amino groups are not always indicated.

Synthesis of 3, 4, 5 and 6[a]

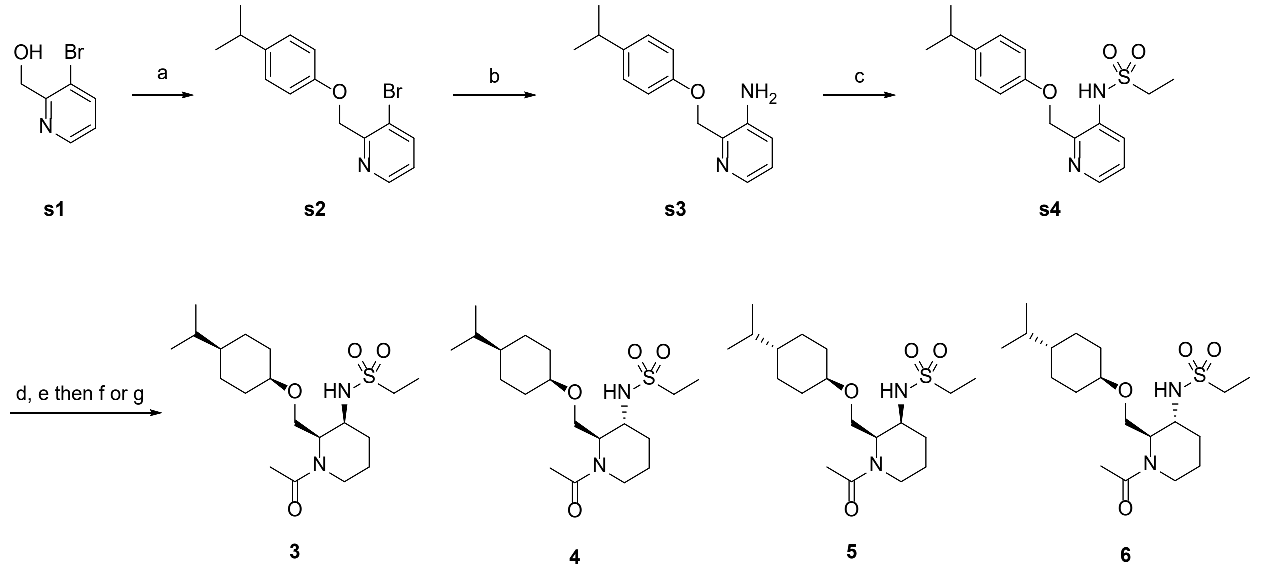

[a]All compounds are racemic mixtures. Reagents and conditions: (a) (1) $SOCl_2$, MeCN, rt, (2) 4-isopropylphenol, K_2CO_3, DMF, rt to 80 °C; (b) (1) diphenylmethanimine, $Pd_2(dba)_3$, XPhos, NaO*t*-Bu, toluene, 80 °C, (2) 2 N HCl, THF, rt; (c) ethanesulfonyl chloride, pyridine, rt; (d) H_2 (44 psi), PtO_2, AcOH, EtOH, 50 °C; (e) Ac_2O, Et_3N, THF, rt; (f) H_2 (1500 psi), 10% Pd/C, AcOH, 100 °C (for **3** and **5**); (g) H_2 (190 psi), 5% Rh/C, EtOH, 170 °C (for **4** and **6**).

3-Bromo-2-((4-isopropylphdenoxy)methyl)pyridine (s2)

To a mixture of **s1** (0.13 g, 6.7 mmol) in MeCN (10 mL) was added a mixture of $SOCl_2$ (0.97 mL, 13.3 mmol) in MeCN (10 mL) over 20 min at rt. The mixture was stirred at rt for 2 h. The mixture was quenched with saturated aqueous $NaHCO_3$ and extracted with EtOAc. The organic layer was separated, washed with saturated aqueous NaCl, dried over anhydrous Na_2SO_4, and concentrated in vacuo. To a solution of the residue and 4-isopropylphenol (0.91 g, 6.7 mmol) in DMF (15 mL) was added K_2CO_3 (1.4 g, 10 mmol) at rt. The mixture was stirred at 80 °C for 2 h. The mixture was quenched with water and extracted with EtOAc. The organic layer was separated, washed with water and saturated aqueous NaCl, dried over anhydrous Na_2SO_4, and

concentrated in vacuo. The residue was purified by column chromatography (basic silica gel, hexane/EtOAc, 100:0 to 1:1) to give **s2** (1.3 g, 4.3 mmol, 64%) as a pale yellow oil. ^{1}H NMR (300 MHz, $CDCl_3$) δ 1.21 (3H, s), 1.24 (3H, s), 2.86 (1H, spt, *J* = 6.9 Hz), 5.27 (2H, s), 6.94−7.01 (2H, m), 7.11−7.21 (3H, m), 7.91 (1H, dd, *J* = 8.1, 1.3 Hz), 8.58 (1H, dd, *J* = 4.5, 1.5 Hz).

2-((4-Isopropylphenoxy)methyl)pyridin-3-amine (s3)

A mixture of **s2** (2.0 g, 6.6 mmol), diphenylmethanimine (1.3 mL, 8.0 mmol), NaO*t*-Bu (0.96 g, 9.9 mmol), XPhos (0.63 g, 1.3 mmol), and $Pd_2(dba)_3$ (0.61 g, 0.66 mmol) in toluene (20 mL) was stirred at 80 °C for 1 h. The mixture was diluted with water and extracted with EtOAc. The organic layer was separated, washed with saturated aqueous NaCl, dried over anhydrous Na_2SO_4, and concentrated in vacuo. The residue was purified by column chromatography (silica gel, hexane/EtOAc, 100:0 to 1:1). A mixture of the residual material and 2 N HCl aqueous solution (5.1 mL, 10 mmol) in THF (15 mL) was stirred at rt for 20 min. The mixture was quenched with saturated aqueous $NaHCO_3$ and extracted with EtOAc. The organic layer was separated, washed with saturated aqueous NaCl, dried over anhydrous Na_2SO_4, and concentrated in vacuo. The residue was purified by column chromatography (basic silica gel, hexane/EtOAc, 100:0 to 7:3) to give **s3** (0.90 g, 3.7 mmol, 74%) as a pale yellow solid. ^{1}H NMR (300 MHz, $CDCl_3$) δ 1.20 (3H, s), 1.22 (3H, s), 2.85 (1H, spt, *J* = 6.9 Hz), 4.21 (2H, brs), 5.24 (2H, s), 6.93−7.01 (3H, m), 7.03−7.10 (1H, m), 7.10−7.17 (2H, m), 8.00 (1H, dd, *J* = 4.5, 1.5 Hz).

***N*-(2-((4-Isopropylphenoxy)methyl)pyridin-3-yl)ethanesulfonamide (s4)**

A mixture of **s3** (1.2 g, 5.0 mmol) and ethanesulfonyl chloride (0.71 mL, 7.5 mmol) in pyridine (15 mL) was stirred at rt overnight. The mixture was concentrated in vacuo, diluted with water,

and extracted with EtOAc. The organic layer was separated, washed with water and saturated aqueous NaCl, dried over anhydrous Na_2SO_4, and concentrated in vacuo. The residue was purified by column chromatography (silica gel, hexane/EtOAc, 19:1 to 1:1). The residual orange oil was treated with activated charcoal in MeOH to give **s4** (1.4 g, 4.2 mmol, 83%) as a pale yellow oil. ^{1}H NMR (300 MHz, $CDCl_3$) δ 1.20 (3H, s), 1.22 (3H, s), 1.34 (3H, t, *J* = 7.4 Hz), 2.85 (1H, spt, *J* = 7.0 Hz), 3.12 (2H, q, *J* = 7.6 Hz), 5.35 (2H, s), 6.92−6.98 (2H, m), 7.11−7.19 (2H, m), 7.27 (1H, dd, *J* = 8.5, 4.7 Hz), 7.85 (1H, brs), 7.95 (1H, dd, *J* = 8.3, 1.5 Hz), 8.34 (1H, dd, *J* = 4.5, 1.5 Hz).

***N*-(*cis*-1-Acetyl-2-(((*cis*-4-isopropylcyclohexyl)oxy)methyl)piperidin-3-yl)ethanesulfonamide (3)**

A mixture of **s4** (1.4 g, 4.2 mmol) and PtO_2 (47 mg, 0.21 mmol) in EtOH (30 mL) and AcOH (20 mL) was hydrogenated under 40 psi at 50 °C overnight. The catalyst was removed by filtration and the filtrate was concentrated in vacuo. The residue was purified by column chromatography (basic silica gel, hexane/EtOAc, 7:3 to 0:100) to give *N*-(*cis*-2-((4-isopropylphenoxy)methyl)piperidin-3-yl)ethanesulfonamide (0.54 g, 1.6 mmol, 38%) as colorless oil. The mixture of *N*-(*cis*-2-((4-isopropylphenoxy)methyl)piperidin-3-yl)ethanesulfonamide (0.54 g, 1.6 mmol), Et_3N (0.66 mL, 4.7 mmol) and Ac_2O (0.22 mL, 2.4 mmol) in THF (7 mL) was stirred at rt overnight. The mixture was quenched with saturated aqueous $NaHCO_3$ and extracted with EtOAc. The organic layer was separated, washed with saturated aqueous NaCl, dried over anhydrous Na_2SO_4, and concentrated in vacuo. The residue was crystallized from EtOAc-hexane to give *N*-(*cis*-1-acetyl-2-((4-isopropylphenoxy)methyl)piperidin-3-yl)ethanesulfonamide (0.52 g, 1.4 mmol, 87%). The mixture of *N*-(*cis*-2-((4-isopropylphenoxy)methyl)piperidin-3-yl)ethanesulfonamide (0.15 g, 0.39 mmol) and 10% Pd-C

(containing 50% water, 75 mg) in AcOH (15 mL) was hydrogenated under 1500 psi at 100 °C for 5 h. The catalyst was removed by filtration and the filtrate was concentrated in vacuo. The residue was diluted with EtOAc and the solution was washed with saturated aqueous $NaHCO_3$, dried over anhydrous Na_2SO_4, and concentrated in vacuo. The residue was purified by a preparative HPLC purification (column, L-Column2 ODS 20 mm ID × 150 mm L; mobile phase A, (10 mM NH_4HCO_3) H_2O/MeCN, 900/100; mobile phase B, (10 mM NH_4HCO_3) H_2O/MeCN, 100/900; flow rate, 20 mL/min) to give **3** (25 mg, 0.064 mmol, 19%) as oil. ^{1}H NMR (600 MHz, DMSO-d_6, rotamer ratio = 75:25) δ 0.79−0.84 (6H, m), 1.00−1.08 (1H, m), 1.11−1.42 (10.75H, m), 1.45−1.57 (1H, m), 1.60−1.73 (2.25H, m), 1.74−1.87 (2H, m), 1.98 (0.75H, s), 2.00 (2.25H, s), 2.50−2.56 (0.75H, m), 2.97−3.16 (2.25H, m), 3.21 (0.25H, dq, *J* = 11.7, 5.9 Hz), 3.34−3.40 (0.75H, m), 3.46−3.51 (1H, m), 3.55−3.62 (1.25H, m), 3.63−3.69 (1H, m), 4.10−4.15 (0.75H, m), 4.25 (0.75H, brdd, *J* = 13.4, 3.9 Hz), 4.80−4.86 (0.25H, m), 7.12 (0.25H, brd, *J* = 7.4 Hz), 7.31 (0.75H, brd, *J* = 6.2 Hz). ^{13}C NMR (151 MHz, DMSO-d_6, the minor rotamer's signals are marked with an asterisk) δ 8.10, 19.41, 19.49, 19.54*, 21.43, 21.76*, 23.05, 23.15, 23.28*, 23.32*, 23.90, 24.90*, 26.14, 26.76*, 28.78, 28.85*, 29.37*, 29.75, 31.93*, 32.00, 34.12, 40.68*, 42.63, 42.66*, 45.55, 45.91*, 50.64*, 51.05*, 51.43, 58.00, 61.09, 62.44*, 72.53*, 72.78, 168.31*, 168.99. MS (ESI/APCI) mass calculated for $[M + H]^+$ ($C_{19}H_{37}N_2O_4S$) requires m/z 388.6, found m/z 389.3.

***N*-(*trans*-1-Acetyl-2-(((*cis*-4-isopropylcyclohexyl)oxy)methyl)piperidin-3-yl)ethanesulfonamide (4)**

A mixture of **s4** (1.4 g, 4.2 mmol) and PtO_2 (47 mg, 0.21 mmol) in EtOH (30 mL) and AcOH (20 mL) was hydrogenated under 44 psi at 50 °C overnight. The catalyst was removed by filtration and the filtrate was concentrated in vacuo. The residue was purified by column chromatography (basic silica gel, hexane/EtOAc, 7:3 to 0:100) to give *N*-(*trans*-2-((4-

isopropylphenoxy)methyl)piperidin-3-yl)ethanesulfonamide (0.19 g, 0.56 mmol, 13%). The mixture of *N*-(*trans*-2-((4-isopropylphenoxy)methyl)piperidin-3-yl)ethanesulfonamide (0.19 g, 0.56 mmol), Et_3N (0.23 mL, 1.7 mmol) and Ac_2O (0.11 mL, 1.1 mmol) in THF (5 mL) was stirred at rt for 2 h. The mixture was quenched with saturated aqueous $NaHCO_3$ and extracted with EtOAc. The organic layer was separated, washed with saturated aqueous NaCl, dried over anhydrous Na_2SO_4, and concentrated in vacuo. The residue was purified by column chromatography (silica gel, hexane/EtOAc, 1:1 to 0:100). The residue was crystallized from EtOAc-hexane to give *N*-(*trans*-1-acetyl-2-((4-isopropylphenoxy)methyl)piperidin-3-yl)ethanesulfonamide (0.20 g, 0.51 mmol, 92%). The mixture of *N*-(*trans*-1-acetyl-2-((4-isopropylphenoxy)methyl)piperidin-3-yl)ethanesulfonamide (0.20 g, 0.51 mmol) and 5% Rh-C (containing 50% water, 0.11 g) in EtOH (10 mL) was hydrogenated under 190 psi at 170 °C for 5 h. The catalyst was removed by filtration and the filtrate was concentrated in vacuo. The residue was purified by a preparative HPLC purification (column, L-Column2 ODS 20 mm ID × 150 mm L; mobile phase A, (10 mM NH_4HCO_3) H_2O/MeCN, 900/100; mobile phase B, (10 mM NH_4HCO_3) H_2O/MeCN, 100/900; flow rate, 20 mL/min) to give **4** (17 mg, 0.044 mmol, 9.7%) as oil. ^1H NMR (600 MHz, DMSO-d_6, rotamer ratio = 67:33) δ 0.81 (4.02H, d, J = 6.6 Hz), 0.83 (1.98H, d, J = 7.0 Hz), 1.00−1.09 (1H, m), 1.13−1.43 (11H, m), 1.54−1.61 (1H, m), 1.62−1.71 (0.67H, m), 1.74 (0.67H, tt, J = 13.2, 3.3 Hz), 1.77−1.87 (2.66H, m), 1.98 (0.99H, s), 2.02 (2.01H, s), 2.55 (0.67H, td, J = 13.0, 2.8 Hz), 2.95 (0.66H, q, J = 7.2 Hz), 3.02−3.08 (1.67H, m), 3.25 (0.33H, dd, J = 9.5, 5.5 Hz), 3.45 (0.67H, dd, J = 9.7, 5.0 Hz), 3.47−3.53 (2H, m), 3.56−3.63 (1H, m), 3.66 (0.33H, brd, J = 7.7 Hz), 4.02−4.07 (0.67H, m), 4.31 (0.67H, brdd, J = 13.4, 2.8 Hz), 4.60 (0.33H, brt, J = 6.6 Hz), 7.33−7.38 (1H, m). ^{13}C NMR (151 MHz, DMSO-d_6, the minor rotamer's signals are marked with an asterisk) δ 8.03, 8.07*, 18.77, 19.04*, 19.40*, 19.48, 21.43, 21.87*, 23.07, 23.15, 23.27*, 25.13, 25.26*, 28.90*, 28.94, 29.48*, 29.51, 31.94*, 31.97, 34.85,

41.73*, 42.59, 45.68, 46.66*, 47.35*, 47.95, 52.57*, 59.12, 64.44, 72.51*, 72.67, 169.14*, 169.58. MS (ESI/APCI) mass calculated for $[M + H]^+$ ($C_{19}H_{37}N_2O_4S$) requires m/z 388.6, found m/z 389.4.

***N*-(*cis*-1-Acetyl-2-(((*trans*-4-isopropylcyclohexyl)oxy)methyl)piperidin-3-yl)ethanesulfonamide (5)**

A mixture of **s4** (1.4 g, 4.2 mmol) and PtO_2 (47 mg, 0.21 mmol) in EtOH (30 mL) and AcOH (20 mL) was hydrogenated under 44 psi at 50 °C overnight. The catalyst was removed by filtration and the filtrate was concentrated in vacuo. The residue was purified by column chromatography (basic silica gel, hexane/EtOAc, 7:3 to 0:100) to give *N*-(*cis*-2-((4-isopropylphenoxy)methyl)piperidin-3-yl)ethanesulfonamide (0.54 g, 1.6 mmol, 38%). The mixture of *N*-(*cis*-2-((4-isopropylphenoxy)methyl)piperidin-3-yl)ethanesulfonamide (0.54 g, 1.6 mmol), Et_3N (0.66 mL, 4.7 mmol), and Ac_2O (0.22 mL, 2.4 mmol) in THF (7 mL) was stirred at rt overnight. The mixture was quenched with saturated aqueous $NaHCO_3$ and extracted with EtOAc. The organic layer was separated, washed with saturated aqueous NaCl, dried over anhydrous Na_2SO_4, and concentrated in vacuo. The residue was crystallized from EtOAc-hexane to give *N*-(*cis*-1-acetyl-2-((4-isopropylphenoxy)methyl)piperidin-3-yl)ethanesulfonamide (0.52 g, 1.4 mmol, 87%). The mixture of *N*-(*cis*-2-((4-isopropylphenoxy)methyl)piperidin-3-yl)ethanesulfonamide (0.15 g, 0.39 mmol) and 10% Pd-C (containing 50% water, 75 mg) in AcOH (15 mL) was hydrogenated under 1500 psi at 100 °C for 5 h. The catalyst was removed by filtration and the filtrate was concentrated in vacuo. The residue was diluted with EtOAc and the solution was washed with saturated aqueous $NaHCO_3$, dried over anhydrous Na_2SO_4, and concentrated in vacuo. The residue was purified by a preparative HPLC purification (column, L-Column2 ODS 20 mm ID × 150 mm L; mobile phase A, (10 mM NH_4HCO_3) H_2O/MeCN,

900/100; mobile phase B, (10 mM NH_4HCO_3) H_2O/MeCN, 100/900; flow rate, 20 mL/min) to give **5** (62 mg, 0.16 mmol, 47%) as oil. ^{1}H NMR (600 MHz, DMSO-d_6, rotamer ratio = 7:3) δ 0.83 (6H, d, J = 6.6 Hz), 0.89−1.01 (3H, m), 1.01−1.12 (2H, m), 1.16−1.21 (3H, m), 1.29−1.43 (1.7H, m), 1.44−1.55 (1.3H, m), 1.59−1.70 (4.3H, m), 1.90−2.01 (5H, m), 2.50−2.55 (0.7H, m), 2.95−3.21 (3.6H, m), 3.31−3.37 (0.7H, m), 3.55 (0.3H, brdd, J = 13.6, 3.7 Hz), 3.63−3.74 (2H, m), 4.04−4.10 (0.7H, m), 4.24 (0.7H, brdd, J = 13.6, 4.0 Hz), 4.80−4.85 (0.3H, m), 7.13 (0.3H, d, J = 7.3 Hz), 7.31 (0.7H, d, J = 7.3 Hz). ^{13}C NMR (151 MHz, DMSO-d_6, the minor rotamer's signals are marked with an asterisk) δ 8.11, 19.73, 19.74, 21.42, 21.73*, 23.93, 24.92*, 26.13, 26.67*, 27.09, 27.12, 27.19*, 27.24*, 31.72*, 31.74, 31.78*, 31.79, 32.01*, 32.04, 34.17, 40.44*, 42.69, 42.76*, 45.56, 46.00*, 50.77*, 50.93*, 51.47, 58.19, 61.35, 62.11*, 77.36*, 78.02, 168.35*, 168.98. MS (ESI/APCI) mass calculated for $[M + H]^+$ ($C_{19}H_{37}N_2O_4S$) requires m/z 388.6, found m/z 389.4.

N-(*trans*-1-Acetyl-2-(((*trans*-4-isopropylcyclohexyl)oxy)methyl)piperidin-3-yl)ethanesulfonamide (6)

A mixture of **s4** (1.4 g, 4.2 mmol) and PtO_2 (47 mg, 0.21 mmol) in EtOH (30 mL) and AcOH (20 mL) was hydrogenated under 44 psi at 50 °C overnight. The catalyst was removed by filtration and the filtrate was concentrated in vacuo. The residue was purified by column chromatography (basic silica gel, hexane/EtOAc, 7:3 to 0:100) to give *N*-(*trans*-2-((4-isopropylphenoxy)methyl)piperidin-3-yl)ethanesulfonamide (0.19 g, 0.56 mmol, 13%). The mixture of *N*-(*trans*-2-((4-isopropylphenoxy)methyl)piperidin-3-yl)ethanesulfonamide (0.19 mg, 0.56 mmol), Et_3N (0.23 mL, 1.67 mmol), and Ac_2O (0.11 mL, 1.12 mmol) in THF (5 mL) was stirred at rt for 2 h. The mixture was quenched with saturated aqueous $NaHCO_3$ and extracted with EtOAc. The organic layer was separated, washed with saturated aqueous NaCl, dried over

anhydrous Na_2SO_4, and concentrated in vacuo. The residue was purified by column chromatography (silica gel, hexane/EtOAc, 1:1 to 0:100). The residue was crystallized from EtOAc-hexane to give *N*-(*trans*-1-acetyl-2-((4-isopropylphenoxy)methyl)piperidin-3-yl)ethanesulfonamide (0.20 g, 0.51 mmol, 92%). The mixture of *N*-(*trans*-1-acetyl-2-((4-isopropylphenoxy)methyl)piperidin-3-yl)ethanesulfonamide (0.20 g, 0.51 mmol) and 5% Rh-C (containing 50% water, 0.11 g) in EtOH (10 mL) was hydrogenated under 190 psi at 170 °C for 5 h. The catalyst was removed by filtration and the filtrate was concentrated in vacuo. The residue was purified by a preparative HPLC purification (column, L-Column2 ODS 20 mm ID × 150 mm L; mobile phase A, (10 mM NH_4HCO_3) H_2O/MeCN, 900/100; mobile phase B, (10 mM NH_4HCO_3) H_2O/MeCN, 100/900; flow rate, 20 mL/min) to give **6** (72 mg, 0.19 mmol, 41%) as oil. ^{1}H NMR (600 MHz, DMSO-d_6, rotamer ratio = 65:35) δ 0.82 (6H, d, J = 6.6 Hz), 0.90−1.00 (3H, m), 1.00−1.11 (2H, m), 1.19 (3H, t, J = 7.3 Hz), 1.34−1.43 (2H, m), 1.52−1.59 (1H, m), 1.61−1.69 (2.65H, m), 1.73 (0.65H, tt, J = 13.4, 3.7 Hz), 1.78−1.84 (0.7H, m), 1.91−2.03 (5H, m), 2.54 (0.65H, td, J = 12.9, 2.4 Hz), 2.91−2.98 (0.7H, m), 2.98−3.08 (1.65H, m), 3.10−3.17 (1H, m), 3.30−3.33 (0.35H, m), 3.46−3.51 (0.65H, m), 3.51−3.66 (2.35H, m), 3.99 (0.65H, brt, J = 6.4 Hz), 4.29 (0.65H, brdd, J = 13.2, 1.8 Hz), 4.57 (0.35H, brt, J = 6.4 Hz), 7.33−7.38 (1H, m). ^{13}C NMR (151 MHz, DMSO-d_6, the minor rotamer's signals are marked with an asterisk) δ 8.04, 8.08*, 18.76, 18.99*, 19.72, 21.42, 21.87*, 24.98, 25.02*, 27.12, 27.18*, 27.20*, 31.74, 31.76*, 31.79*, 31.86, 31.91, 32.02*, 34.93, 41.57*, 42.66, 42.68*, 45.71, 46.74*, 47.18*, 47.89, 52.52*, 59.19, 64.47*, 64.70, 77.67*, 77.99, 169.15*, 169.54. MS (ESI/APCI) mass calculated for $[M + H]^+$ ($C_{19}H_{37}N_2O_4S$) requires m/z 388.6, found m/z 389.4.

Synthesis of 10, 13, 13a and 13b[a]

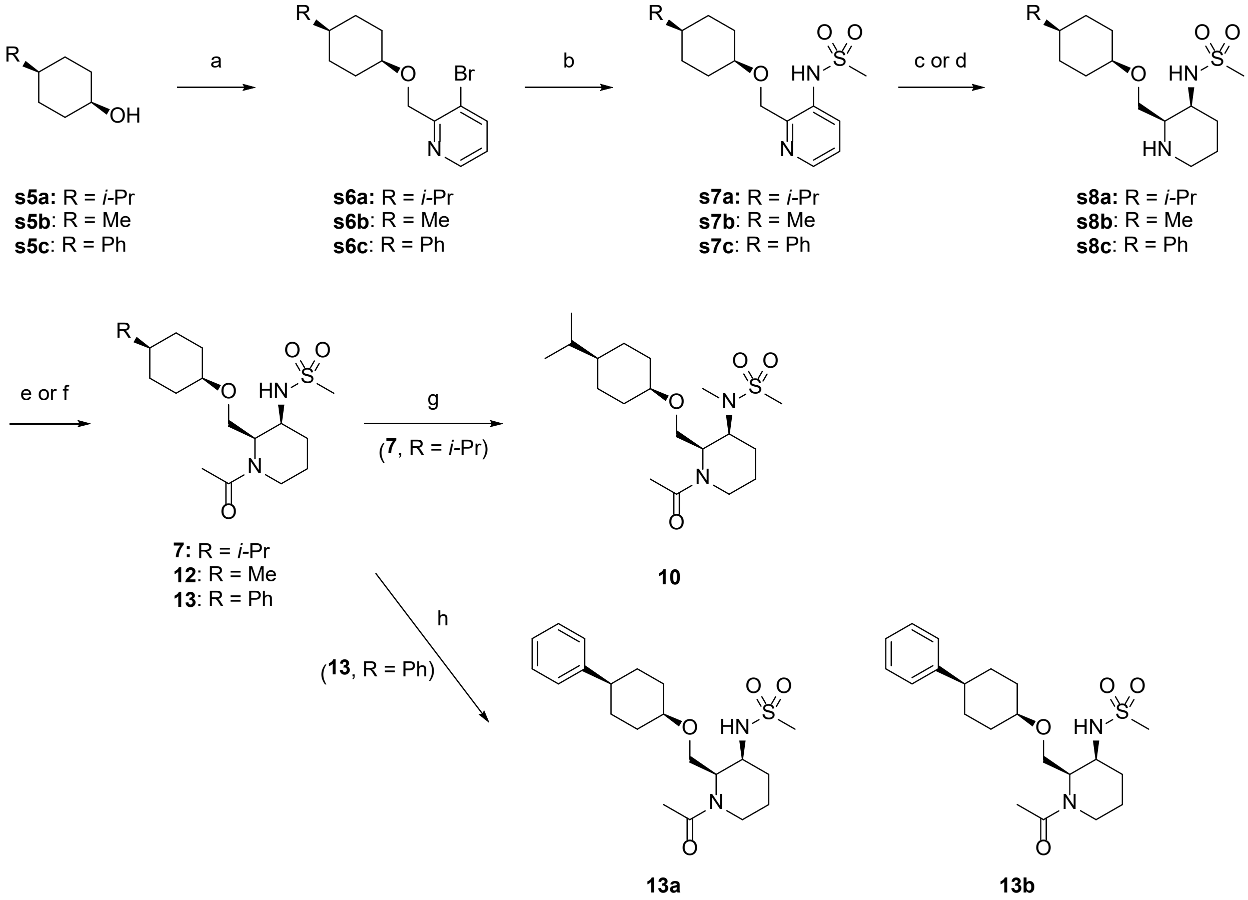

[a] Reagents and conditions: (a) bromo-2-(bromomethyl)pyridine, 60% NaH in oil, THF, 0 °C–rt; (b) methanesulfonamide, $Pd_2(dba)_3$, *t*-BuXPhos, Cs_2CO_3, THF, 120 °C (microwave); (c) H_2 (87 psi), PtO_2, AcOH, MeOH, 50 °C (for **s8a** and **s8b**); (d) H_2 (balloon), PtO_2, AcOH, MeOH, rt (for **s8c**); (e) AcCl, DIEA, DMA, rt (for **7**); (f) Ac_2O, Et_3N, THF, 0 °C–rt (for **12** and **13**); (g) **7**, 60% NaH in oil, MeI, DMA, 0 °C; (h) **13**, chiral column separation.

3-Bromo-2-(((*cis*-4-isopropylcyclohexyl)oxy)methyl)pyridine (s6a)

To a suspension of 60% NaH in oil (7.0 g, 0.18 mol) in THF (80 mL) was added **s5a** (20 g, 0.14 mol) at rt. The mixture was stirred at rt overnight. To the mixture was added a mixture of bromo-2-(bromomethyl)pyridine (18 g, 70 mmol) in THF (80 mL) and the mixture was stirred at rt

overnight. The mixture was quenched with saturated aqueous NH_4Cl and extracted with EtOAc. The organic layer was separated, washed with saturated aqueous NaCl, dried over anhydrous Na_2SO_4, and concentrated in vacuo. The residue was purified by column chromatography (basic silica gel, hexane/EtOAc, 100:0 to 4:1) to give **s6a** (17 g, 55 mmol, 79%) as a colorless oil. ^{1}H NMR (300 MHz, $CDCl_3$) δ 0.86 (6H, d, *J* = 6.8 Hz), 0.98−1.14 (1H, m), 1.34−1.54 (7H, m), 1.90−2.03 (2H, m), 3.65−3.78 (1H, m), 4.69 (2H, s), 7.11 (1H, dd, *J* = 8.1, 4.7 Hz), 7.86 (1H, dd, *J* = 8.1, 1.3 Hz), 8.53 (1H, dd, *J* = 4.7, 1.3 Hz).

3-Bromo-2-(((*cis*-4-methylcyclohexyl)oxy)methyl)pyridine (s6b)

The title compound was prepared in 30% yield (0.30 g, 35 mmol) as a colorless oil from **s5b** (1.2 g, 11 mmol) using the procedure analogous to that described for the synthesis of **s6a**. ^{1}H NMR (300 MHz, $CDCl_3$) δ 0.90 (3H, d, *J* = 6.0 Hz), 1.26−1.62 (7H, m), 1.84−2.07 (2H, m), 3.67 (1H, dd, *J* = 4.7, 2.3 Hz), 4.70 (2H, s), 7.11 (1H, dd, *J* = 8.1, 4.7 Hz), 7.86 (1H, dd, *J* = 8.0, 1.4 Hz), 8.54 (1H, dd, *J* = 4.7, 1.5 Hz).

3-Bromo-2-(((*cis*-4-phenylcyclohexyl)oxy)methyl)pyridine (s6c)

The title compound was prepared in 85% yield (84 g, 0.24 mmol) as a pale yellow oil from **s5c** (51 g, 0.29 mol) using the procedure analogous to that described for the synthesis of **s6a**. ^{1}H NMR (300 MHz, $CDCl_3$) δ 1.56 (2H, d, *J* = 3.0 Hz), 1.62−1.71 (2H, m), 1.87−2.01 (2H, m), 2.11−2.21 (2H, m), 2.45−2.70 (1H, m), 3.79−3.89 (1H, m), 4.74 (2H, s), 7.09−7.20 (2H, m), 7.20−7.26 (3H, m), 7.27−7.32 (1H, m), 7.88 (1H, dd, *J* = 8.0, 1.5 Hz), 8.47−8.59 (1H, m).

***N*-(2-(((*cis*-4-Isopropylcyclohexyl)oxy)methyl)pyridin-3-yl)methanesulfonamide (s7a)**

The mixture of **s6a** (3.0 g, 9.6 mmol), methanesulfonamide (1.1 g, 12 mmol), XPhos (0.41 g, 0.96 mmol), $Pd_2(dba)_3$ (0.44 g, 0.48 mmol), Cs_2CO_3 (4.7 g, 14 mmol), and THF (40 mL) was heated at 120 °C for 20 min under microwave irradiation. The mixture was passed through a pad of Celite and the filtrate was extracted with EtOAc. The organic layer was separated, washed with water and saturated aqueous NaCl, dried over anhydrous $MgSO_4$, and concentrated in vacuo. The residue was purified by column chromatography (silica gel, hexane/EtOAc, 19:1 to 1:1) to give **s7a** (2.3 g, 7.1 mmol, 73%) as an orange oil. ^{1}H NMR (300 MHz, $CDCl_3$) δ 0.86 (3H, s), 0.88 (3H, s), 1.00−1.16 (1H, m), 1.22−1.56 (7H, m), 1.89−2.08 (2H, m), 3.03 (3H, s), 3.63−3.77 (1H, m), 4.81 (2H, s), 7.25 (1H, dd, *J* = 8.5, 4.7 Hz), 7.93 (1H, dd, *J* = 8.3, 1.5 Hz), 8.29 (1H, dd, *J* = 4.5, 1.5 Hz), 8.80 (1H, s).

***N*-(2-(((*cis*-4-Methylcyclohexyl)oxy)methyl)pyridin-3-yl)methanesulfonamide (s7b)**

The title compound was prepared in 57% yield (0.63 g, 2.1 mmol) as a yellow oil from **s6b** (1.1 g, 3.7 mmol) using the procedure analogous to that described for the synthesis of **s7a**. ^{1}H NMR (300 MHz, $CDCl_3$) δ 0.87−0.97 (3H, m), 1.18−1.62 (10H, m), 1.85−2.00 (2H, m), 3.66−3.75 (1H, m), 4.78−4.84 (2H, m), 7.21−7.25 (1H, m), 7.88−7.98 (1H, m), 8.25−8.32 (1H, m), 8.78 (1H, s).

***N*-(2-(((*cis*-4-Phenylcyclohexyl)oxy)methyl)pyridin-3-yl)methanesulfonamide (s7c)**

The title compound was prepared in 75% yield (2.3 g, 6.5 mmol) as a yellow solid from **s6c** (3.0 g, 8.7 mmol) using the procedure analogous to that described for the synthesis of **s7a**. ^{1}H NMR (300 MHz, $CDCl_3$) δ 1.60−1.88 (6H, m), 2.11 (2H, brs), 2.46−2.67 (1H, m), 3.05 (3H, s), 3.82 (1H, t, *J* = 2.8 Hz), 4.86 (2H, s), 7.25 (3H, s), 7.27−7.35 (3H, m), 7.94 (1H, dd, *J* = 8.3, 1.5 Hz), 8.30 (1H, dd, *J* = 4.9, 1.5 Hz), 8.79 (1H, s).

***N*-(*cis*-2-(((*cis*-4-Isopropylcyclohexyl)oxy)methyl)piperidin-3-yl)methanesulfonamide (s8a)**

A mixture of **s7a** (2.3 g, 7.1 mmol) and PtO_2 (80 mg, 0.35 mmol) in MeOH (15 mL) and AcOH (15 mL) was hydrogenated under 87 psi at 50 °C for 16 h. The catalyst was removed by filtration and the filtrate was concentrated in vacuo. The residue was diluted with EtOAc, washed with saturated aqueous $NaHCO_3$ and saturated aqueous NaCl, and concentrated in vacuo. The residue was purified by column chromatography (basic silica gel, hexane/EtOAc, 100:0 to 2:3) to give **s8a** (1.6 g, 4.9 mmol, 69%) as a pale yellow oil. ^{1}H NMR (300 MHz, $CDCl_3$) δ 0.84 (3H, s), 0.87 (3H, s), 0.96−1.11 (1H, m), 1.19−2.03 (14H, m), 2.66 (1H, td, J = 11.8, 2.8 Hz), 2.86 (1H, ddd, J = 7.8, 4.4, 2.1 Hz), 2.97 (3H, s), 3.04 (1H, dt, J = 11.5, 2.4 Hz), 3.33 (1H, dd, J = 9.4, 7.9 Hz), 3.46 (2H, dd, J = 9.4, 4.5 Hz), 3.59 (1H, brs), 5.36 (1H, d, J = 7.2 Hz).

***N*-(*cis*-2-(((*cis*-4-Methylcyclohexyl)oxy)methyl)piperidin-3yl)methanesulfonamide (s8b)**

The title compound was prepared in 70% yield (443 mg, 1.5 mmol) as a yellow solid from **s7b** (620 mg, 2.1 mmol) using the procedure analogous to that described for the synthesis of **s8a**. ^{1}H NMR (300 MHz, $CDCl_3$) δ 0.84−0.95 (3H, m), 1.14−1.33 (2H, m), 1.35−1.87 (11H, m), 1.90−2.02 (1H, m), 2.57−2.74 (1H, m), 2.80−2.89 (1H, m), 2.97 (3H, s), 3.01−3.11 (1H, m), 3.25−3.39 (1H, m), 3.42−3.52 (2H, m), 3.55−3.66 (1H, m), 5.20−5.49 (1H, m).

***N*-(*cis*-2-(*cis*-4-Phenylcyclohexyloxymethyl)-piperidin-3-yl)-methanesulfonamide (s8c)**

A mixture of **s7c** (1.0 g, 2.8 mmol) and PtO_2 (63 mg, 0.28 mmol) in MeOH (24 mL) and AcOH (8 mL) was hydrogenated under balloon pressure at 50 °C for 4 h. The catalyst was removed by

filtration and the filtrate was concentrated in vacuo. The residue was purified by column chromatography (basic silica gel, hexane/EtOAc, 7:3 to 0:100) to afford **s8c** (0.69 g, 1.9 mmol, 47%) as a waxy solid. ^{1}H NMR (400 MHz, $CDCl_3$) δ 1.49−1.78 (9H, m), 1.95−2.02 (3H, m), 2.48−2.56 (1H, m), 2.65−2.71 (1H, m), 2.87−2.90 (1H, m), 2.96 (3H, s), 3.02−3.05 (1H, m), 3.36 (1H, t, J = 9.2 Hz), 3.48−3.51 (1H, m), 3.58−3.62 (2H, m), 5.37 (1H, d, J = 7.6 Hz), 7.15−7.28 (5H, m).

***N*-(*cis*-1-Acetyl-2-(((*cis*-4-isopropylcyclohexyl)oxy)methyl)piperidin-3-yl)methanesulfonamide (7)**

To a mixture of **s8a** (0.10 g, 0.30 mmol) and DIEA (0.079 mL, 0.45 mmol) in DMA (5 mL) was added AcCl (35 mg, 0.45 mmol) at rt. The mixture was stirred at rt overnight. The mixture was poured into water and extracted with EtOAc. The organic layer was separated, washed with 1 N HCl aqueous solution and saturated aqueous NaCl, dried over anhydrous $MgSO_4$, and concentrated in vacuo. The residue was purified by column chromatography (silica gel, hexane/EtOAc, 1:9 to 0:100) to give **7** (0.11 g, 0.29 mmol, 98%) as an amorphous solid. ^{1}H NMR (300 MHz, $CDCl_3$) δ 0.76−0.95 (6H, m), 0.99−2.26 (17H, m), 2.44−3.17 (4H, m), 3.38−6.33 (7H, m). MS (ESI/APCI) mass calculated for $[M + H]^+$ ($C_{18}H_{35}N_2O_4S$) requires m/z 374.5, found m/z 375.2.

***N*-(*cis*-1-Acetyl-2-(((*cis*-4-isopropylcyclohexyl)oxy)methyl)piperidin-3-yl)-*N*-methylmethanesulfonamide (10)**

To a mixture of **7** (0.11 g, 0.30 mmol) in DMA (10 mL) was added 60% NaH in oil (18 mg, 0.45 mmol) at 0 °C. After being stirred at 0 °C for 10 min, MeI (0.028 mL, 0.45 mmol) was

added to the reaction mixture at 0 °C. The mixture was stirred at 0 °C for 1 h. The mixture was neutralized with 1 N HCl aqueous solution at 0 °C and extracted with EtOAc. The organic layer was separated, washed with water and saturated aqueous NaCl, dried over anhydrous $MgSO_4$, and concentrated in vacuo. The residue was purified by column chromatography (silica gel, hexane/EtOAc, 17:3 to 0:100) to give **10** (0.11 g, 0.28 mmol, 94%) as a white solid. ^{1}H NMR (300 MHz, $CDCl_3$) δ 0.75−0.94 (6H, m), 0.98−2.23 (16H, m), 2.25−3.07 (7H, m), 3.36−4.31 (6H, m), 4.50−4.83 (1H, m). MS (ESI/APCI) mass calculated for $[M + H]^+$ ($C_{19}H_{37}N_2O_4S$) requires m/z 388.6, found m/z 389.4.

***N*-(*cis*-1-Acetyl-2-(((*cis*-4-methylcyclohexyl)oxy)methyl)piperidin-3-yl)methanesulfonamide (12)**

To a mixture of **s8b** (50 mg, 0.16 mmol) and Et_3N (28 mg, 0.28 mmol) in THF (5 mL) was added Ac_2O (25 mg, 0.25 mmol) at 0 °C. The mixture was stirred at rt for 15 h. The mixture was poured into water at rt and was extracted with EtOAc. The organic layer was separated, washed with saturated aqueous NaCl, dried over anhydrous $MgSO_4$, and concentrated in vacuo. The residue was purified by column chromatography (silica gel, EtOAc/MeOH, 100:0 to 0:100) to give **12** (52 mg, 0.15 mmol, 92%) as a pale yellow oil. ^{1}H NMR (300 MHz, $CDCl_3$) δ 0.80−0.98 (3H, m), 1.09−1.33 (3H, m), 1.35−1.66 (8H, m), 1.71−1.91 (4H, m), 1.95−3.18 (6H, m), 3.39−6.29 (6H, m). MS (ESI/APCI) mass calculated for $[M + H]^+$ ($C_{16}H_{31}N_2O_4S$) requires m/z 346.5, found m/z 347.1.

***N*-(*cis*-1-Acetyl-2-(((*cis*-4-phenylcyclohexyl)oxy)methyl)piperidin-3-yl)methanesulfonamide (13)**

To a mixture of **s8c** (0.25 g, 0.68 mmol) and Et_3N (0.24 mL, 1.7 mmol) in THF (5 mL) was added Ac_2O (0.096 mL, 1.0 mmol) at rt. The reaction mixture was stirred at rt overnight. The mixture was quenched with saturated aqueous $NaHCO_3$ and extracted with EtOAc three times. The organic layer was separated, washed with brine, dried over anhydrous $MgSO_4$, and concentrated in vacuo. The residue was purified by column chromatography (basic silica gel, hexane/EtOAc, 7:3 to 1:4) to give **13** (0.25 g, 0.61 mmol, 89%) as a colorless oil. ^1H NMR (300 MHz, $CDCl_3$) δ 1.55-2.64 (17H, m), 2.95−3.15 (4H, m), 3.54−3.71 (3H, m), 3.86−4.02 (1H, m), 4.29−6.26 (2H, m), 7.08−7.38 (5H, m). MS (ESI/APCI) mass calculated for $[M + H]^+$ ($C_{21}H_{33}N_2O_4S$) requires m/z 408.6, found m/z 409.1.

***N*-(*rel*-(*2R*,*3S*)-1-Acetyl-2-(((*cis*-4-phenylcyclohexyl)oxy)methyl)piperidin-3-yl)methanesulfonamide (13a**)

Resolution of the enantiomers of **13** was carried out chromatographically using a Chiralcel OJ 50 mm ID × 500 mmL column (hexane/EtOH, 600:400) at 60 mL/min. Resolution of **13** (0.25 g, 0.60 mmol) provided **13a** (0.11 g, 0.26 mmol, 43%) as an amorphous solid. Analytical HPLC analysis carried out on a 4.6 mm ID × 250 mmL Chiralcel OJ column (hexane/EtOH, 600:400) at a flow rate of 0.5 mL/min indicated that **13a** was of >99.9% enantiomeric excess (ee). ^1H NMR (300 MHz, $CDCl_3$) δ 1.55-2.64 (17H, m), 2.95−3.15 (4H, m), 3.54−3.71 (3H, m), 3.86−4.02 (1H, m), 4.29−6.26 (2H, m), 7.08−7.38 (5H, m). MS (ESI/APCI) mass calculated for $[M + H]^+$ ($C_{21}H_{33}N_2O_4S$) requires m/z 408.6, found m/z 409.2.

***N*-(*rel*-(*2S*,*3R*)-1-Acetyl-2-(((*cis*-4-phenylcyclohexyl)oxy)methyl)piperidin-3-**

yl)methanesulfonamide (13b)

Resolution of the enantiomers of **13** was carried out chromatographically using a Chiralcel OJ 50 mm ID × 500 mmL column (hexane/EtOH, 600:400) at 60 mL/min. Resolution of **13** (0.25 g, 0.60 mmol) provided **13b** (0.11 g, 0.26 mmol, 43%) as an amorphous solid. Analytical HPLC analysis carried out on a 4.6 mm ID × 250 mm L Chiralcel OJ column (hexane/EtOH, 600:400) at a flow rate of 0.5 mL/min indicated that **13b** was of 99.8% ee. ^{1}H NMR (300 MHz, $CDCl_3$) δ 1.55-2.64 (17H, m), 2.95−3.15 (4H, m), 3.54−3.71 (3H, m), 3.86−4.02 (1H, m), 4.29−6.26 (2H, m), 7.08−7.38 (5H, m). MS (ESI/APCI) mass calculated for $[M + H]^+$ ($C_{21}H_{33}N_2O_4S$) requires m/z 408.6, found m/z 409.1.

Synthesis of 8, 9 and 11[a]

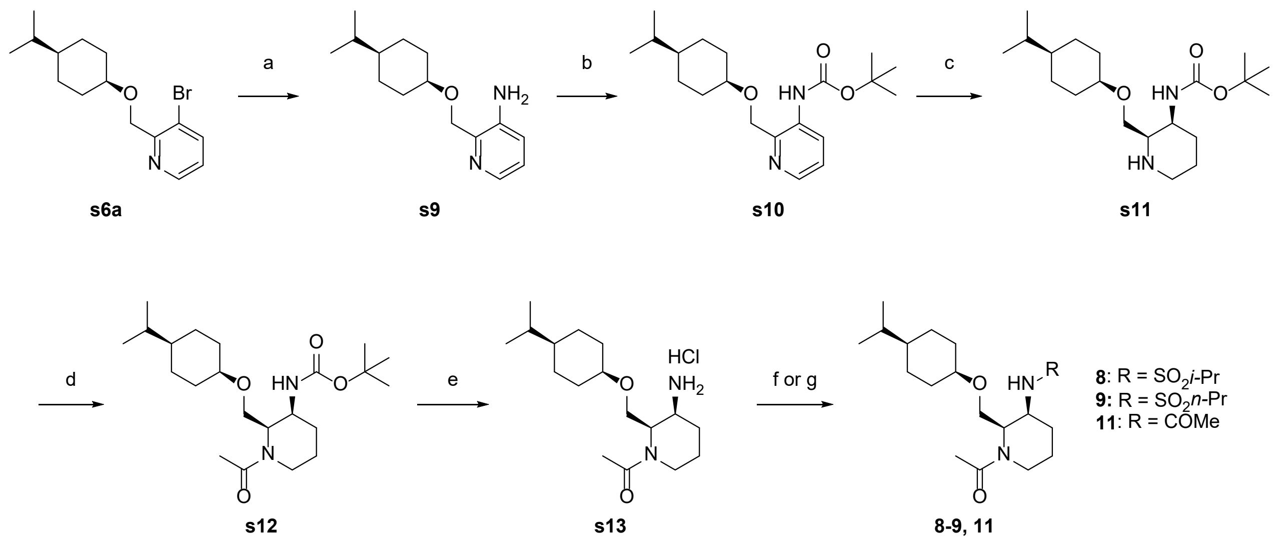

[a]All compounds are racemic mixtures. Reagents and conditions: (a) (1) diphenylmethanimine, $Pd_2(dba)_3$, XPhos, NaO*t*-Bu, toluene, 80 °C; (2) 2 N HCl, THF, rt; (b) Boc_2O, 1.9 M NaHMDS in THF, THF, rt; (c) H_2 (87 psi), PtO_2, AcOH, MeOH, rt; (d) AcCl, DIEA, DMA, rt; (e) 4 N HCl in EtOAc, EtOAc, rt; (f) RSO_2Cl (for **8** and **9**), DBU, THF, rt; (g) AcCl, DIEA, DMA, rt (for **11**).

2-(((*cis*-4-Isopropylcyclohexyl)oxy)methyl)pyridin-3-amine (s9)

The title compound was prepared in 77% yield (1.3 g, 5.0 mmol) as a white solid from **s6a** (3.1 g, 10 mmol) using the procedure analogous to that described for the synthesis of **s3**. ^1H NMR (300 MHz, $CDCl_3$) δ 0.80−0.90 (6H, m), 0.98−1.16 (1H, m), 1.25−1.52 (7H, m), 1.88−2.02 (2H, m), 3.64 (1H, brs), 4.40 (2H, brs), 4.65−4.74 (2H, m), 6.91−6.99 (1H, m), 7.03 (1H, dd, *J* = 7.9, 4.5 Hz), 7.93 (1H, dd, *J* = 4.5, 1.5 Hz).

***tert*-Butyl (2-(((*cis*-4-isopropylcyclohexyl)oxy)methyl)pyridin-3-yl)carbamate (s10)**

To a mixture of **s9** (0.66 g, 3,0 mmol) in THF (20 mL) was added 1.9 M NaHMDS in THF (3.3 mL, 6.3 mmol) at rt and the resulting mixture was stirred at rt for 10 min. Boc_2O (0.70 mL, 3.0 mmol) was added to the mixture at rt, and the resulting mixture was stirred at rt overnight. The mixture was poured into water and extracted with EtOAc. The organic layer was separated, washed with saturated aqueous NaCl, dried over anhydrous $MgSO_4$, and concentrated in vacuo. The residue was purified by column chromatography (silica gel, hexane/EtOAc, 100:0 to 1:1) to give **s10** (0.55 g, 1.6 mmol, 53%) as a pale yellow oil. ^1H NMR (300 MHz, DMSO-d_6) δ 0.83 (6H, d, J = 6.8 Hz), 0.95−1.54 (17H, m), 1.78−1.92 (2H, m), 3.58−3.68 (1H, m), 4.69 (2H, s), 7.24−7.38 (1H, m), 8.09−8.22 (2H, m), 8.51−8.65 (1H, m).

***tert*-Butyl (*cis*-2-(((*cis*-4-isopropylcyclohexyl)oxy)methyl)piperidin-3-yl)carbamate (s11)**

A mixture of **s10** (0.54 g, 1.6 mmol) and PtO_2 (18 mg, 0.080 mmol) in MeOH (10 mL) and AcOH (10 mL) was hydrogenated under 87 psi at rt overnight. The catalyst was removed by filtration and the filtrate was neutralized with 1 N NaOH aqueous solution at 0 °C and extracted with EtOAc. The organic layer was separated, washed with saturated aqueous NaCl, dried over anhydrous Na_2SO_4, and concentrated in vacuo. The residue was purified by column chromatography (basic silica gel, hexane/EtOAc, 100:0 to 3:2) to give **s11** (0.29 g, 0.82 mmol, 53%) as a white solid. ^1H NMR (300 MHz, $CDCl_3$) δ 0.81−0.90 (6H, m), 0.93−1.13 (1H, m), 1.17−1.55 (16H, m), 1.56 (1H, brs), 1.65 (5H, brs), 1.84 (3H, brs), 2.57−2.75 (1H, m), 2.85 (1H, d, J = 9.1 Hz), 2.98−3.12 (1H, m), 3.20 (1H, t, J = 9.3 Hz), 3.38−3.52 (2H, m), 3.71 (1H, d, J = 7.2 Hz), 5.37 (1H, d, J = 9.1 Hz).

***tert*-Butyl (*cis*-1-acetyl-2-(((*cis*-4-isopropylcyclohexyl)oxy)methyl)piperidin-3-yl)carbamate (s12)**

To a mixture of **s11** (0.36 g, 1.0 mmol) and DIEA (0.21 mL, 1.2 mmol) in DMA (10 mL) was added AcCl (0.085 mL, 1.2 mmol) at rt. The mixture was stirred at rt overnight. The mixture was poured into water and extracted with EtOAc. The organic layer was separated, washed with 1 N HCl aqueous solution and saturated aqueous NaCl, dried over anhydrous $MgSO_4$, and concentrated in vacuo. The residue was purified by column chromatography (silica gel, hexane/EtOAc, 9:1 to 0:100) to give **s12** (0.29 g, 0.73 mmol, 73%) as a white amorphous solid. ^{1}H NMR (300 MHz, DMSO-d_6) δ 0.67−0.89 (6H, m), 0.96−2.09 (25H, m), 2.93−3.68 (6H, m), 4.14−4.95 (2H, m), 6.67−7.12 (1H, m).

1-(*cis*-3-Amino-2-(((*cis*-4-isopropylcyclohexyl)oxy)methyl)piperidin-1-yl)ethanone hydrochloride (s13)

To a mixture of **s12** (0.31 mg, 0.77 mmol) in EtOAc (2 mL) was added 4 N HCl in EtOAc (0.96 mL, 3.9 mmol) at rt. The mixture was stirred at rt under N_2 for 1 h. The solvent was evaporated in vacuo to give **s13** (0.25 g, 0.75 mmol, 98%) as an amorphous solid. ^{1}H NMR (300 MHz, DMSO-d_6) δ 0.70−0.91 (6H, m), 0.96−1.50 (8H, m), 1.66−2.11 (8H, m), 2.22−2.47 (3H, m), 2.93−3.19 (1H, m), 3.43−3.77 (3H, m), 4.22−5.11 (2H, m), 7.84−8.11 (2H, m).

***N*-(*cis*-1-Acetyl-2-(((*cis*-4-isopropylcyclohexyl)oxy)methyl)piperidin-3-yl)propane-2-sulfonamide (8)**

To a mixture of **s13** (44 mg, 0.13 mmol) in THF (5 mL) was added DBU (80 mg, 0.53 mmol) at

0 °C. After being stirred at 0 °C for 10 min, propane-2-sulfonyl chloride (38 mg, 0.26 mmol) was added to the reaction mixture at rt. The mixture was stirred at rt overnight. After concentration in vacuo, the residue was purified by column chromatography (silica gel, hexane/EtOAc, 80:20 to 0:100) to give **8** (15 mg, 0.037 mmol, 28%) as oil. ^{1}H NMR (300 MHz, $CDCl_3$) δ 0.86 (6H, d, J = 6.8 Hz), 0.99−2.35 (23H, m), 2.45−3.26 (2H, m), 3.35−6.04 (7H, m). MS (ESI/APCI) mass calculated for $[M + H]^+$ ($C_{20}H_{39}N_2O_4S$) requires m/z 402.6, found m/z 403.1.

***N*-(*cis*-1-Acetyl-2-(((*cis*-4-isopropylcyclohexyl)oxy)methyl)piperidin-3-yl)propane-1-sulfonamide (9)**

To a mixture of **s13** (48 mg, 0.14 mmol) in THF (5 mL) was added DBU (87 mg, 0.57 mmol) at 0 °C. After being stirred at 0 °C for 10 min, propane-1-sulfonyl chloride (41 mg, 0.29 mmol) was added to the reaction mixture at rt. The mixture was stirred at rt overnight. After concentration in vacuo, the residue was purified by column chromatography (silica gel, hexane/EtOAc, 80:20 to 0:100) to give **9** (34 mg, 0.084 mmol, 59%) as oil. ^{1}H NMR (300 MHz, $CDCl_3$) δ 0.86 (6H, d, J = 6.8 Hz), 0.97−2.29 (22H, m), 2.50−3.20 (3H, m), 3.28−6.19 (7H, m). MS (ESI/APCI) mass calculated for $[M + H]^+$ ($C_{20}H_{39}N_2O_4S$) requires m/z 402.6, found m/z 403.2.

***N*-(*cis*-1-Acetyl-2-(((*cis*-4-isopropylcyclohexyl)oxy)methyl)piperidin-3-yl)acetamide (11)**

To a mixture of **s13** (83 mg, 0.25 mmol) and DIEA (0.087 mL, 0.50 mmol) in DMA (4 mL) was added AcCl (0.027 mL, 0.38 mmol) at rt. The mixture was stirred at rt overnight. The mixture was poured into water and extracted with EtOAc. The organic layer was separated, washed with saturated aqueous NaCl, dried over $MgSO_4$, and concentrated in vacuo. The residue was purified by column chromatography (silica gel, EtOAc/MeOH, 100:0 to 7:3) to give **11** (63 mg, 0.19

mmol, 74%) as a colorless oil. ^{1}H NMR (300 MHz, $CDCl_3$) δ 0.87 (6H, d, J = 6.8 Hz), 1.00−1.16 (1H, m), 1.16−2.26 (19H, m), 2.41−3.70 (4H, m), 3.74−4.10 (2H, m), 4.18−4.64 (1H, m), 4.88−6.62 (1H, m). MS (ESI/APCI) mass calculated for $[M + H]^+$ ($C_{19}H_{35}N_2O_3$) requires m/z 338.5, found m/z 339.3.

Synthesis of 14, 15, 16, 17 and 18[a]

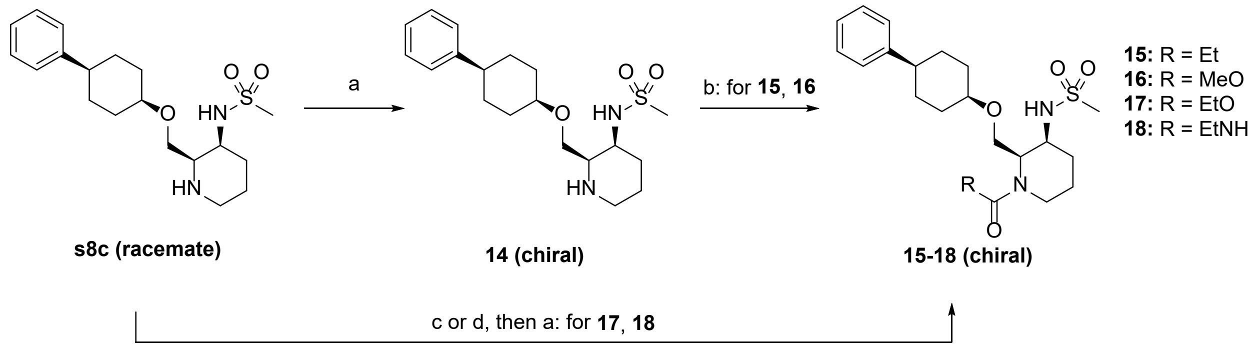

s8c (racemate) **14 (chiral)** **15-18 (chiral)**

[a]Reagents and conditions: (a) chiral column separation; (b) RCOCl, Et_3N, THF, rt (for **15** and **16**); (c) ethyl chlorocarbonate, DIEA, THF, rt (for **17**); (d) isocyanatoethane, Et_3N, THF, 0 °C−rt (for **18**).

***N*-(*rel*-(2*R*,3*S*)-2-(((*cis*-4-Phenylcyclohexyl)oxy)methyl)piperidin-3-yl)methanesulfonamide (14)**

Resolution of the enantiomers of **s8c** was carried out chromatographically using a Chiralpak AD 50 mm ID × 500 mmL column (hexane/EtOH/Et_2NH, 700:300:1) at 60 mL/min. Resolution of **s8c** (4.3 g, 12 mmol) provided **14** (1.6 g, 4.4 mmol, 38%) as oil. Analytical HPLC analysis carried out on a 4.6 mm ID × 250 mm L Chiralpak AD column (hexane/EtOH/Et_2NH, 700:300:1) at a flow rate of 0.5 mL/min indicated that **14** was of >99.9% ee. ^{1}H NMR (600 MHz, DMSO-d_6) δ 1.31−1.38 (1H, m), 1.45−1.55 (5H, m), 1.63−1.84 (4H, m), 1.87−1.98 (3H, m), 2.50−2.56 (2H,

m), 2.79 (1H, td, J = 6.6, 2.6 Hz), 2.87−2.92 (1H, m), 2.93 (3H, s), 3.28−3.33 (1H, m), 3.34−3.39 (1H, m), 3.50 (1H, brs), 3.57 (1H, brquin, J = 3.0 Hz), 6.70 (1H, brs), 7.16 (1H, t, J = 6.9 Hz), 7.21 (2H, d, J = 7.4 Hz), 7.27 (2H, t, J = 7.6 Hz). MS (ESI/APCI) mass calculated for $[M + H]^+$ ($C_{19}H_{31}N_2O_3S$) requires m/z 366.5, found m/z 367.1.

***N*-(*rel*-(2*R*,3*S*)-2-(((*cis*-4-Phenylcyclohexyl)oxy)methyl)-1-propionylpiperidin-3-yl)methanesulfonamide (15)**

To a mixture of **14** (58 mg, 0.16 mmol) and Et_3N (0.044 mL, 0.32 mmol) in THF (3 mL) was added propionyl chloride (0.021 mL, 0.24 mmol) at rt. The mixture was stirred at rt. The mixture was quenched with water and extracted with EtOAc. The organic layer was separated, washed with saturated aqueous NaCl, dried over anhydrous Na_2SO_4, and concentrated in vacuo. The residue was purified by column chromatography (silica gel, hexane/EtOAc, 2:3 to 0:100) to give **15** (67 mg, 0.16 mmol, 100%) as a colorless oil. ^{1}H NMR (300 MHz, $CDCl_3$) δ 1.15 (3H, t, J = 7.4 Hz), 1.54−1.63 (6H, m), 1.70−1.85 (4H, m), 1.92−2.09 (3H, m), 2.24−2.67 (3H, m), 3.00 (3H, s), 3.53 (1H, d, J = 5.7 Hz), 3.67 (2H, brs), 3.85−4.09 (1H, m), 4.26−4.70 (1H, m), 5.04−5.61 (1H, m), 6.15 (1H, d, J = 7.6 Hz), 7.13−7.26 (3H, m), 7.27−7.35 (2H, m). MS (ESI/APCI) mass calculated for $[M + H]^+$ ($C_{22}H_{35}N_2O_4S$) requires m/z 422.6, found m/z 423.2.

Methyl (2*R*,3*S*)-3-((methylsulfonyl)amino)-2-(((*cis*-4-phenylcyclohexyl)oxy)methyl)piperidine-1-carboxylate (16)

To a mixture of **14** (58 mg, 0.16 mmol) and Et_3N (0.044 mL, 0.32 mmol) in THF (3 mL) was added methyl chlorocarbonate (0.024 mL, 0.32 mmol) at rt. The mixture was stirred at rt overnight. The mixture was quenched with water and extracted with EtOAc. The organic layer was separated, washed with saturated aqueous NaCl, dried over anhydrous Na_2SO_4, and

concentrated in vacuo. The residue was purified by column chromatography (silica gel, hexane/EtOAc, 1:1 to 0:100) to give **16** (64 mg, 0.15 mmol, 95%) as a colorless oil. Crystallization of **16** (1.8 g, 4.1 mmol) from EtOH-H_2O gave **16** (1.7 g, 3.9 mmol, 95%) as a white solid. ^{1}H NMR (600 MHz, DMSO-d_6) δ 1.40−1.55 (5H, m), 1.56−1.73 (5H, m), 1.87 (1H, brd, J = 13.2 Hz), 1.96 (1H, brd, J = 13.6 Hz), 2.44−2.57 (1H, m), 2.83 (1H, brs), 2.95 (3H, s), 3.40 (1H, brs), 3.53−3.62 (5H, m), 3.73 (1H, brt, J = 9.7 Hz), 3.84 (1H, brs), 4.47 (1H, brs), 7.15 (1H, brt, J = 7.2 Hz), 7.18 (1H, brs), 7.19 (2H, brd, J = 8.1 Hz), 7.27 (2H, brt, J = 7.4 Hz). ^{13}C NMR (151 MHz, DMSO-d_6, the minor rotamer's signals are marked with an asterisk) δ24.05, 24.39*, 26.00, 26.17*, 27.60*, 27.79, 28.68, 30.15*, 37.54, 38.13*, 39.91, 42.99, 51.01, 52.07, 53.90*, 54.49, 61.48, 61.89*, 71.68, 125.68, 126.51, 128.14, 147.34, 155.27*, 156.08. MS (ESI/APCI) mass calculated for $[M + H]^+$ ($C_{21}H_{33}N_2O_5S$) requires m/z 424.6, found m/z 425.2. mp 113 °C. Anal. Calcd for $C_{21}H_{32}N_2O_5S$: C, 59.41; H, 7.60; N, 6.60. Found: C, 59.45; H, 7.59; N, 6.55. $[\alpha]_D^{20}$ +16.3 (c 0.1, $CHCl_3$)

Ethyl *rel*-(*2R*,*3S*)-3-((methylsulfonyl)amino)-2-(((*cis*-4-phenylcyclohexyl)oxy)methyl)piperidine-1-carboxylate (17)

To a mixture of **s8c** (50 mg, 0.14 mmol) and DIEA (0.071 mL, 0.41 mmol) in THF (3 mL) was added ethyl chlorocarbonate (0.039 mL, 0.41 mmol) at rt. The mixture was stirred at rt for 1.5 h. The mixture was quenched with water and extracted with EtOAc. The organic layer was separated, washed with saturated aqueous NaCl, dried over anhydrous Na_2SO_4, and concentrated in vacuo. The residue was purified by column chromatography (silica gel, hexane/EtOAc, 1:1 to 0:100) to give the racemic compound. Chiral separation (Chiralcel OJ-H 20 mm ID × 250 mmL column (CO_2/MeOH/Et_2NH, 900:100:1) at 60 mL/min) gave **17** (23 mg, 0.052 mmol, 37%) as a colorless oil. Analytical HPLC analysis carried out on a 4.6 mm ID × 250 mmL Chiralcel OJ-H

column (hexane/EtOH, 600:400) at a flow rate of 1.0 mL/min indicated that **17** was of >99.9% ee. ^{1}H NMR (300 MHz, $CDCl_3$) δ 1.20−1.32 (3H, m), 1.55−1.88 (9H, m), 1.95−2.16 (3H, m), 2.45−2.61 (1H, m), 2.70−2.88 (1H, m), 2.99 (3H, s), 3.52−3.73 (3H, m), 3.90−4.07 (2H, m), 4.08−4.25 (2H, m), 4.63 (1H, d, *J* = 3.8 Hz), 6.04 (1H, brs), 7.14−7.26 (3H, m), 7.29 (2H, d, *J* = 7.2 Hz). MS (ESI/APCI) mass calculated for $[M + H]^+$ ($C_{22}H_{35}N_2O_5S$) requires m/z 438.6, found m/z 439.2.

***rel*-(*2R*,*3S*)-*N*-Ethyl-3-((methylsulfonyl)amino)-2-(((*cis*-4-phenylcyclohexyl)oxy)methyl)piperidine-1-carboxamide (18)**

To a mixture of **s8c** (0.28 mg, 0.76 mmol) and Et_3N (0.32 mL, 2.3 mmol) in THF (2 mL) was added isocyanatoethane (81 mg, 1.2 mmol) at 0 °C. The mixture was stirred at rt overnight, quenched with water, and extracted with EtOAc. The organic layer was washed with saturated aqueous NaCl, dried over anhydrous Na_2SO_4, and concentrated in vacuo. The residue was purified by column chromatography (silica gel, hexane/EtOAc, 1:1 to 0:100) to give the racemic compound. Chiral separation (Chiralpak IC 50 mm ID × 500 mmL column (hexane/*i*-PrOH, 200:800) at 60 mL/min) gave **18** (0.15 g, 0.35 mmol, 46%) as an amorphous solid. Analytical HPLC analysis carried out on a 4.6 mm ID × 250 mmL Chiralpak IC column (hexane/*i*-PrOH, 200:800) at a flow rate of 0.5 mL/min indicated that **18** was of >99.9% ee. ^{1}H NMR (300 MHz, $CDCl_3$) δ 1.14 (3H, t, *J* = 7.3 Hz), 1.60−1.74 (8H, m), 1.98−2.09 (4H, m), 2.46−2.61 (1H, m), 2.74−2.91 (1H, m), 3.01 (3H, s), 3.26 (2H, qd, *J* = 7.2, 5.4 Hz), 3.50−3.70 (3H, m), 3.71−3.83 (1H, m), 3.93 (1H, dd, *J* = 9.3, 7.6 Hz), 4.47−4.60 (1H, m), 4.72 (1H, t, *J* = 5.1 Hz), 5.75 (1H, d, *J* = 7.7 Hz), 7.17-7.32 (5H, m). MS (ESI/APCI) mass calculated for $[M + H]^+$ ($C_{22}H_{36}N_3O_4S$) requires m/z 437.6, found m/z 438.3.

ABBREVIATIONS USED

DMF, *N*,*N*-dimethylformamide; THF, tetrahydrofuran; dba, dibenzylideneacetone; XPhos, dicyclohexyl(2’,4’,6’-triisopropyl-[1,1’-biphenyl]-2-yl)phosphine; Pd/C, palladium on carbon; Rh/C, rhodium on carbon; *t*-BuXPhos, di-*tert*-butyl(2’,4’,6’-triisopropyl-[1,1’-biphenyl]-2-yl)phosphine; DIEA, *N*-ethyl-*N*-isopropylpropan-2-amine; DMA, *N*,*N*-dimethylacetamide; NaHMDS, sodium bis(trimethylsilyl)amide; DBU, 2,3,4,6,7,8,9,10-octahydropyrimido[1,2-a]azepine

Calcium flux assay (in vitro agonistic activity of OX2R and OX1R)[1]

hOX1R/CHO-K1 cells and hOX2R/CHO-K1 cells suspended in Ham's F-12 medium (Fujifilm Wako Pure Chemical Co.) supplemented with 100 U/mL penicillin-streptomycin and 10% FBS were plated in blackwalled clear-bottomed 384-well plates (Corning, NY, USA) at 10000 cells/well and in black-walled clear-bottomed 96-well plates (Corning) at 45000 cells/well, respectively. The plated cells were grown overnight at 37 °C in the presence of 5% CO2. The following day, the medium was removed, and the cells were incubated with assay buffer (HBSS with 20mM HEPES, pH 7.4, and 0.1% fatty acid free BSA) containing 2.5 µg/mL Fluo-4 AM (Cat.# F312, Dojindo Laboratories, Kumamoto, Japan), 1.25mM probenecid (Dojindo Laboratories), and 0.08% Pluronic F127 (Dojindo Laboratories) for 30 min at 37 °C in the presence of 5% CO2. After incubation, the cells were stimulated with test compounds in the assay buffer. Calcium mobilization was measured using an FDSS/µCELL (Hamamatsu Photonics K.K., Shizuoka, Japan). The responses to 0.11% DMSO and 100 nM OX-A were used to represent the 0% and 100% responses, respectively.

Transcellular transport study using a transporter-expression system

Human MDR1-expressing LLC-PK1 cells were cultured as reported previously with minor modifications.[2] The transcellular transport study was performed as reported previously.[2] In brief, the cells were grown for 7 days in an HTS Transwell 96-well permeable support (pore size, 0.4 μm; surface area, 0.143 cm^2) with a polyethylene terephthalate membrane (Corning Life Sciences, Lowell, MA, USA) at a density of 1.125×10^5 cells/well. The cells were preincubated with M199 at 37 °C for 30 min. Subsequently, transcellular transport was initiated by the addition of M199 either to apical compartments (75 μL) or to the basolateral compartments (250 μL) containing 10 μM digoxin, 200 μM lucifer yellow as a marker for the monolayer tightness, and 10 μM test compounds and then terminated by the removal of each assay plate after 2 h. The aliquots (25 μL) in the opposite compartments were mixed with acetonitrile containing alprenolol and diclofenac as an internal standard and then centrifuged. The compound concentrations in the supernatant were measured by LC−MS/MS. The apparent permeability (P_{app}) of test compounds in the receiver wells was determined, and the efflux ratio for the MDR1 membrane permeability test was calculated using the following equation:

Efflux ratio = $P_{\mathrm{app,BtoA}}/P_{\mathrm{app,AtoB}}$

where $P_{\mathrm{app,AtoB}}$ is the apical-to-basal passive permeability−surface area product and $P_{\mathrm{app,BtoA}}$ is the basal-to-apical passive permeability−surface area product.

Measurement of plasma and brain concentration of compound 16 in mice

The aliquots of the brain homogenate were mixed with acetonitrile containing internal standard. The mixtures were centrifuged. The supernatants were diluted with solvents for LC-MS/MS (mobile phase A: 10 mM ammonium formate/formic acid (100/0.2, v/v), mobile phase B: acetonitrile/formic acid (100/0.2, v/v)). The diluted solutions were injected into LC-MS/MS (API5000, AB Sciex, CA) equipped with Shimadzu Shim-pack XR-ODS (2.2 μm, 2.0 × 30 mm, Shimadzu Corporation, Japan) at 50 °C. Analyst software TM (version 1.4.2) was used for data acquisition and processing.

Ethics Statement. The care and use of the animals and the experimental protocols used in this research were approved by the Experimental Animal Care and Use Committee of Takeda Pharmaceutical Company Limited.

Evaluation of wakefulness time measurement of compound 16 in mice

ICR mice were obtained from CLEA Japan Inc. (Tokyo, Japan). Mice were housed in groups of 4 per cage in a light controlled room (12 h light/dark cycle with lights on at 07:00 h). Food and tap water were provided *ad libitum*. The care and use of the animals and the experimental protocols in this study were approved by the Institutional Animal Care and Use Committee of Takeda Pharmaceutical Company Limited. Implantation of EEG electrodes was performed as described previously.[1] Compound **16** at 3 mg/kg suspended in 0.5% methylcellulose saline was administered subcutaneously to mice at zeitgeber time 5 (ZT5) in a volume of 10 mL/kg body weight, and then EEG was recorded. EEG analysis was performed with data collected during the first 3 hr after administration of Compound **16**. Data were presented as the mean + standard error of the mean (S.E.M.). The statistical significance between two groups was determined by paired t-test with significance set at $P < 0.05$.

Ethics Statement. The care and use of the animals and the experimental protocols used in this research were approved by the Experimental Animal Care and Use Committee of Takeda Pharmaceutical Company Limited.

^{1}H NMR and ^{13}C NMR charts of compound 16

1) 1H NMR

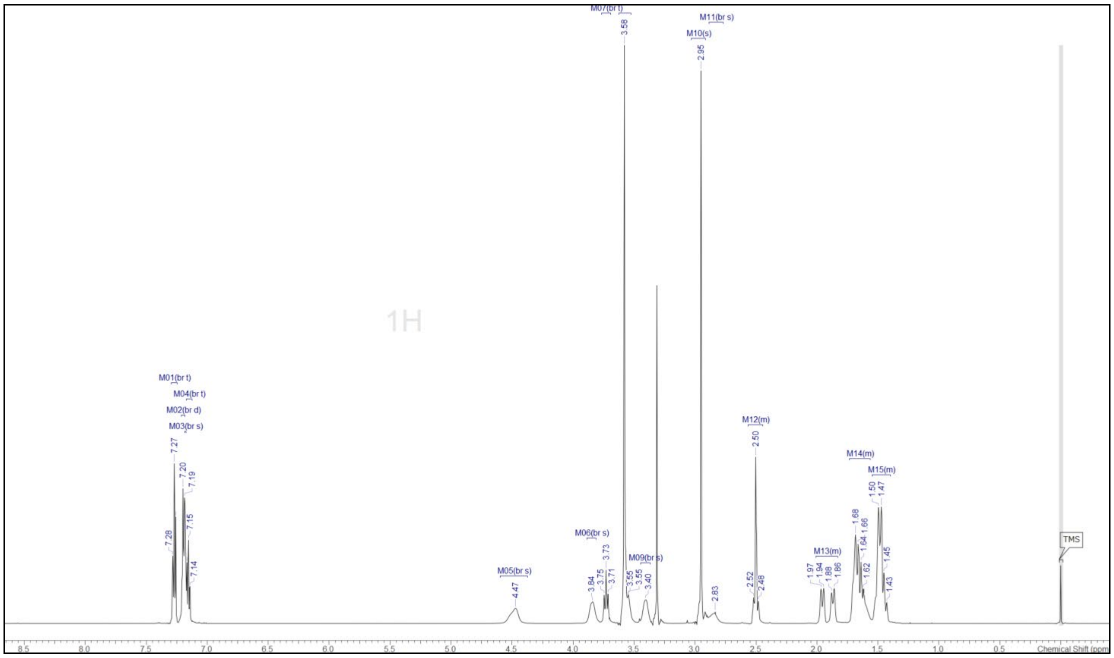

2) 13C NMR

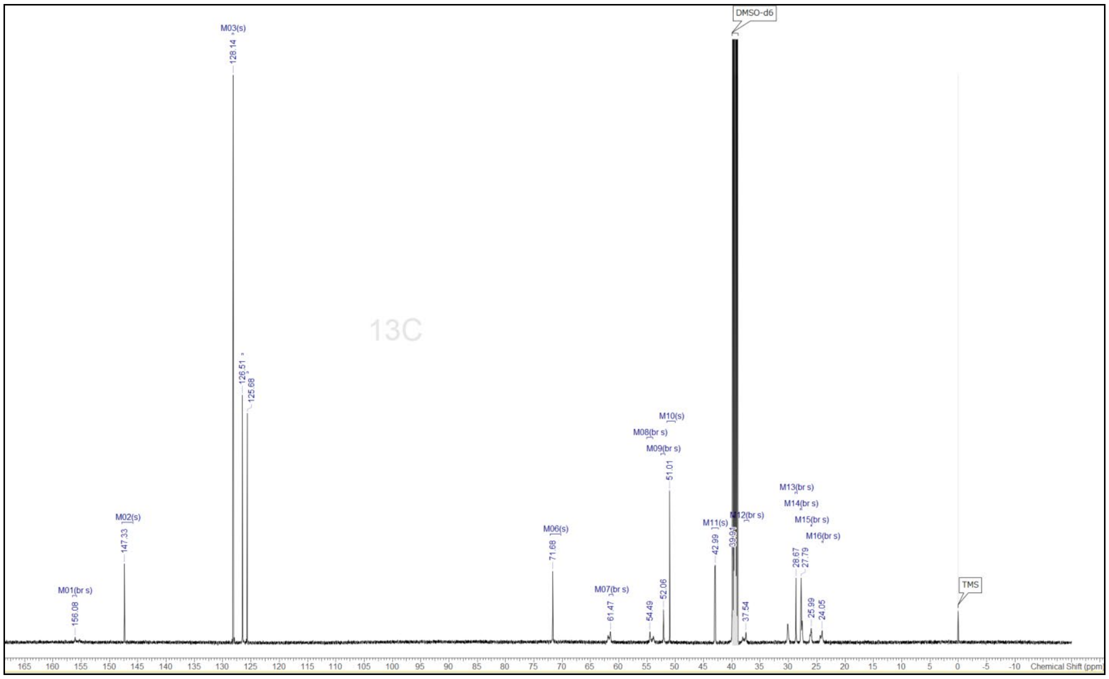

Single-crystal X-ray structure analysis of compound 16

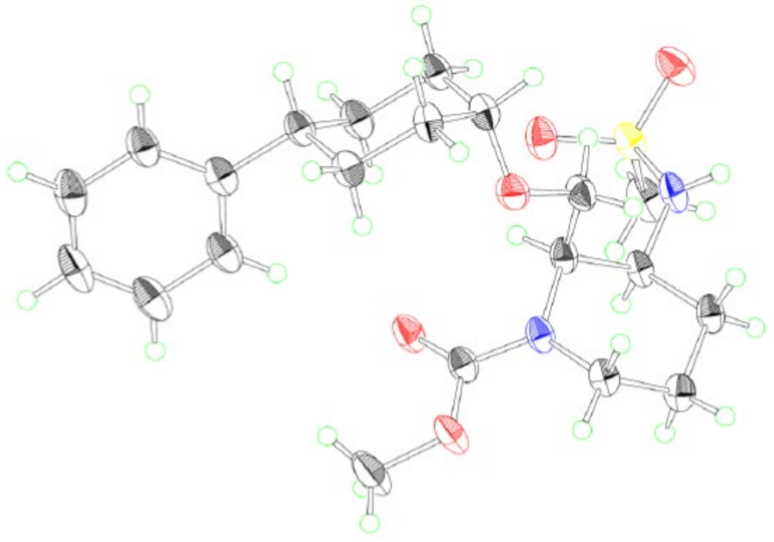

Figure S1. ORTEP of compound **16**, thermal ellipsoids are drawn at 50% probability.

Crystal data for compound **16**: $C_{21}H_{32}N_2O_5S$ *MW* = 424.55; crystal size, 0.24 x 0.12 x 0.05 mm; colourless, plate; monoclinic, space group $P2_1$, a = 9.66931(13) Å, b = 11.39460(14) Å, c = 10.36070(10) Å, $\alpha = \gamma = 90°$, β = 106.5990(12)°, V = 1093.95(2) Å^3, Z = 2, Dx = 1.289 g/cm^3, T = 100 K, μ = 1.599 mm^{-1}, λ = 1.54184 Å, R_1 = 0.0525, wR_2 = 0.1427, S = 1.046, Flack Parameter[3] = 0.01(2).

All measurements were made on a Rigaku XtaLAB P200 diffractometer using multi-layer mirror monochromated Cu-Kα radiation. The structure was solved by direct methods with SHELXT-2018/2[4] and was refined using full-matrix least-squares on F^2 with SHELXL-2018/3.[5] All non-H atoms were refined with anisotropic displacement parameters.

CCDC 2111149 for compound **16** contains the supplementary crystallographic data for this paper. These data can be obtained free of charge from The Cambridge Crystallographic Data Centre via http://www.ccdc.cam.ac.uk/structures.

References

(1) Yukitake, H.; Fujimoto, T.; Ishikawa, T.; Suzuki, A.; Shimizu, Y.; Rikimaru, K.; Ito, M.; Suzuki, M.; Kimura, H. TAK-925, an orexin 2 receptor-selective agonist, shows robust wake-promoting effects in mice. *Pharmacol. Biochem. Behav.* **2019**, *187*, 172794.

(2) Takeuchi, T.; Yoshitomi, S.; Higuchi, T.; Ikemoto, K.; Niwa, S.; Ebihara, T.; Katoh, M.; Yokoi, T.; Asahi, S. Establishment and characterization of the transformants stably-expressing MDR1 derived from various animal species in LLC-PK1. *Pharm. Res.* **2006**, *23*, 1460−1472.

(3) Parsons, S.; Flack, H. D.; Wagner, T. Use of intensity quotients and differences in absolute structure refinement. *Acta Cryst. B,* **2013**, *69*, 249-259.

(4) Sheldrick, G.M. A SHELXT: Integrating space group determination and structure solution. *Acta Cryst. A*, **2014**, *70*, C1437.

(5) Sheldrick, G.M. A short history of SHELX. *Acta Cryst. A*, **2008**, *64*, 112-122.
